# Activated CD8^+^ T cell extracellular vesicles prevent tumour progression by targeting of lesional mesenchymal cells

**DOI:** 10.1038/s41467-018-02865-1

**Published:** 2018-01-30

**Authors:** Naohiro Seo, Yoshitaka Shirakura, Yoshiro Tahara, Fumiyasu Momose, Naozumi Harada, Hiroaki Ikeda, Kazunari Akiyoshi, Hiroshi Shiku

**Affiliations:** 1 Department of Immuno-Gene Therapy, Mie University Graduate School of Medicine, Edobashi, Tsu, Mie 514-8507 Japan; 2 ERATO Bio-Nanotransporter Project, Japan Science and Technology Agency (JST), Kyoto, 615-8530 Japan; 3 Department of Applied Chemistry, Graduate School of Engineering, Kyushu University, Moto-oka, Fukuoka, 819-0395 Japan; 4 Department of Oncology, Nagasaki University Graduate School of Biomedical Sciences, Sakamoto, Nagasaki, 852-8523 Japan; 5 Department of Polymer Chemistry, Graduate School of Engineering, Katsura Int’tech Center, Kyoto University, Nishikyo-ku, Kyoto, 615-8530 Japan

## Abstract

Fibroblastic tumour stroma comprising mesenchymal stem cells (MSCs) and cancer-associated fibroblasts (CAFs) promotes the invasive and metastatic properties of tumour cells. Here we show that activated CD8^+^ T cell-derived extracellular vesicles (EVs) interrupt fibroblastic stroma-mediated tumour progression. Activated CD8^+^ T cells from healthy mice transiently release cytotoxic EVs causing marked attenuation of tumour invasion and metastasis by apoptotic depletion of mesenchymal tumour stromal cells. Infiltration of EV-producing CD8^+^ T cells is observed in neovascular areas with high mesenchymal cell density, and tumour MSC depletion is associated with preferential engulfment of CD8^+^ T cell EVs in this setting. Thus, CD8^+^ T cells have the capacity to protect tumour progression by EV-mediated depletion of mesenchymal tumour stromal cells in addition to their conventional direct cytotoxicity against tumour cells.

## Abstract (web summary)

Immune cells have an important role in tumour progression. Here, the authors show that extracellular vesicles from activated CD8^+^ T cells attenuate tumour progression by depletion of mesenchymal tumour stromal cells.

## Introduction

A wide variety of cells including immune cells release diverse types of extracellular vesicles (EVs) of endosome and plasma membrane origin known as exosomes and microvesicles with sizes 40–150 nm and 100–1000 nm, respectively^1,2^. Physiologically active substances including various proteins and nucleic acids (e.g., cytokines, mRNAs, microRNAs [miRNAs]) are found in EVs and they act as central mediators of the regulation of neighbouring and distant-recipient cells with incorporated EVs^3,4^.

Dendritic cell (DC)-derived EVs directly enhance the antigen-specific responses of CD4^+^ and CD8^+^ T cells and participate in the activation of NK cells^5^. EV miRNAs from T cells are transferred into DCs in an antigen-specific manner^6^. In addition, it has been reported that regulatory T cell-derived EVs act as suppressors against pathogenic Th1 responses in an miRNA-dependent manner^7^. These findings indicate that the parent cell functions are inherited by EVs in part via miRNAs. Activated CD8^+^ T cells have a central role in the exclusion of tumour cells by direct interaction with tumour antigen peptides in the context of MHC class I molecules^8^, suggesting that the derived EVs are cytotoxic against tumour cells. Recently, it has been reported that CD8^+^ T cells transmigrate into tumour lesions by releasing granzyme B that mediates remodelling of the basement membrane of tumour blood vessels^9^. This report suggested that CD8^+^ T cells have a tumoricidal function that involves an unknown mechanism in addition to direct tumour cell killing, e.g., cytotoxicity against tumour stromal cells, modulation of tumour angiogenesis and/or vascularisation, intrusion into tumour or tumour stromal areas and prevention of tumour invasion and metastasis by acquisition of mesenchymal-like properties in part in an EV-mediated fashion.

Tumour stroma is formed by various infiltrating and locally differentiated cell populations, e.g., tumour-associated macrophages (TAMs: F4/80^+^), DCs (CD11c^+^), myeloid-derived suppressor cells (MDSCs: CD11b^+^ and granulocyte receptor [Gr]-1^+^), cancer-associated fibroblasts (CAFs: fibroblast markers [e.g., murine ER-TR7^+^] and α-smooth muscle actin [SMA]^+^), and mesenchymal stem cells (MSCs: platelet-derived growth factor-α [PDGFRα: CD140a]^+^ and stem cell antigen [Sca]-1^+^)^10^ along with tumour angiogenesis (Sca-1^+^ and CD31^+^)^11^ to fill gaps in tumour areas with extracellular matrix proteins^12,13^. During the malignant transformation process, tumour cells acquire mesenchymal-like features that enable metastatic migration into blood vessels and invasive spreading through the tumour capsule. This process is mainly caused by transforming growth factor (TGF)-β-mediated complicated molecular mechanisms^12,14,15^ and EV-dependent actions between tumour cells and tumour stromal cells such as MSCs and CAFs^2,16–21^.

In this study, we investigated whether EVs from activated CD8^+^ T cells are involved in the regulation of tumour progression by intratumoural (i.t.) administration, and found that activated CD8^+^ T cells from healthy mice interrupt tumour invasion and metastasis by depleting tumoural mesenchymal cells.

## Results

### Depletion of mesenchymal stroma in CD8 EV-treated tumour

To clarify the involvement of EVs from activated CD8^+^ T cells in direct tumour cell killing, various cultured tumour cell lines were mixed with EVs. Splenocytes from mutated (m) ERK2 peptide (a H-2K^d^-restricted epitope for CMS5a tumour cells)-specific TCR gene-transgenic DUC18 mice^22^ or BALB/c mice splenocytes were cultured, and the supernatants were used as a source of EVs from tumour-specific or nonspecific CD8^+^ T cells, respectively (Supplementary Fig. 1a: DUC18 CD8 EV or BALB CD8 EV). As shown in Supplementary Figs. 1b–d, 2, 3a, b, 10a and 12d, DUC18 CD8 EVs and BALB CD8 EVs failed to modulate various tumour cell lines. Next, we investigated in detail the role of activated CD8^+^ T cell EVs against tumour tissues.

Growth of subcutaneous CMS5a tumours (1.0–1.2 cm tumour diameter) was significantly attenuated in DUC18 CD8 EV- and BALB CD8 EV-treated groups by i.t. administration compared to BALB CD4 EV (from CD8^+^ cell-depleted BALB/c splenocytes)-, CMS5a EV- or hPBMC EV-treated groups (Supplementary Fig. 4a). Spheroid formation observed after cultivation (24 h) of CMS5a tumour suspensions disappeared in DUC18 CD8 EV-treated cases (Supplementary Fig. 4b). Growth of CT26 on BALB/c mice or B16 on B6 mice was also attenuated by i.t. treatment with DUC18 CD8 EVs (Supplementary Fig. 4c). Furthermore, the attenuated growth of DUC18 CD8 EV- and BALB CD8 EV-treated CMS5a was visualised by Ki-67 staining (Supplementary Fig. 4d). Collectively, these results indicate that activated CD8^+^ T cells, but not activated CD4^+^ T cells, tumour cells or human CD8^+^ T cells, release EVs that downregulate tumour growth and reduce in vitro spheroid formation.

**Fig. 1:**
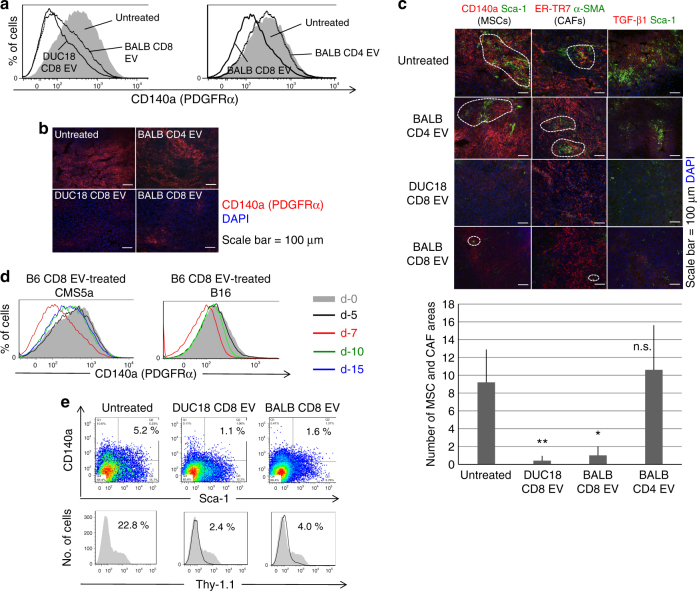
Depletion of tumour mesenchymal stroma by i.t. treatment with CD8^+^ T cell EVs. Expressions of CD140a in a CMS5a tumour (*n* = 4 per group) treated intratumorally with/without BALB CD8 EVs, DUC18 CD8 EVs or BALB CD4 EVs (10 μg [5–7 × 10^8^ vesicles]/tumour) are shown as histogram data obtained by flow cytometry (**a**) and representative photos of fluorescent immunohistochemistry (**b**). Each photo is a representative of 4–5 photos. **c** CMS5a tumour on day 3 after treatment with the indicated EVs (10 μg [5–7 × 10^8^ vesicles]/tumour) were sectioned and dual stained with CD140a- and Sca-1-specific mAbs, ER-TR7- and α-smooth muscle actin-specific mAbs, or TGF-β- and Sca-1-specific mAbs. Each photo is a representative of 5–6 photos. The dotted circles express the overlapping areas of PE and FITC fluorescence. The number of overlapping areas of DUC18 CD8 EVs (**) and BALB CD8 EVs (*)-treated groups were significantly different from the untreated group with *p* < 0.01 and *p *< 0.05, respectively (n.s., not significant; error bars indicate SEM). The data were analysed by a two-tailed unpaired Student *t*-test. **d** EVs (10 μg [5–7 × 10^8^ vesicles]/tumour) prepared from day 5, 7, 10 or 15 culture medium of both TRP-2 peptide and gd100 peptide-stimulated B6 splenocytes were injected into subcutaneous CMS5a or B16 tumours. Three days after EV treatment, the CD140a expression in tumour cell suspensions was examined by flow cytometric analysis. **e** CMS5a cells were inoculated subcutaneously into Thy-1.1^+^ bone-derived MSC-chimeric BALB/c mice (*n* = 4 mouse per group). DUC18 CD8 EVs or BALB CD8 EVs (10 μg [5–7 × 10^8^ vesicles]/tumour) were injected into day 12 subcutaneous CMS5a tumours. 3 days after EV treatment, the % of CD140a^+^ and Sca-1^+^ cells and transferred Thy 1.1^+^ cells was examined by flow cytometry

Next, we examined the fluctuation of cell populations reported as regulators of tumour progression. Although the proportion of CD4^+^ cells, CD8^+^ cells, macrophages, DCs or MDSCs were not changed in EV-treated CMS5a tumours (Supplementary Fig. 5), the expression of CD140a and TGF-β was markedly decreased in association with the disappearance of MSC and CAF areas on day 3 after i.t. administration of DUC18 CD8 and BALB CD8 EVs (Figs. 1a−c). The time-course study of functional EV production was performed using CD8^+^ T cell EVs from both B16 melanoma-associated both TRP-2 peptide- and gp100 peptide-stimulated B6 splenocytes (B6 CD8 EVs). The B16-specific CD8^+^ T cell population gradually increased during cultivation and comprised 95 and 3%, respectively, of the CD8^+^ populations on day 15 (Supplementary Fig. 6). Notably, the reduction of CD140a expression peaked when EVs from the 7-day cultured CD8^+^ T cells were applied to unrelated CMS5a as well as to relevant B16 tumours (Fig. 1d). There was no difference in tumour growth or tumour cell populations including MSCs and CAFs after intratumoural treatment of EVs from cultured CMS5a-bearing BALB/c CD8^+^ splenocytes (BALB TB CD8 EVs) (Supplementary Figs. 5, 7a–c and 10a), indicating the importance of the biological conditions as well as the culture period for CD8^+^ T cells to release functional EVs.

To confirm the anti-mesenchymal stromal nature of CD8^+^ T cell-derived EVs in vivo, MSCs were cultured from crushed femur bones of Thy-1.1^+^ BALB/c congenic mice (Supplementary Fig. 8a–c), and Thy-1.1^+^ MSC-chimeric BALB/c mice were prepared by transferring cultured Thy-1.1^+^ MSCs into irradiated Thy-1.2^+^ BALB/c mice (Supplementary Fig. 9). When DUC18 CD8 EVs and BALB CD8 EVs were injected into subcutaneous CMS5a tumours in Thy-1.1^+^ MSC-chimeric BALB/c mice, nearly all tumour Thy-1.1^+^ cells, including 5–7% of transferred CD140a^+^ Sca-1^+^ MSCs and 22% of MSC-differentiated cells, disappeared (Fig. 1e). In conclusion, CD8^+^ T cells from healthy mice can produce EVs that destroy mesenchymal tumour stroma in the same species without H-2 restriction.

### Reduced viability of cultured MSC by treatment with CD8 EV

**Fig. 2:**
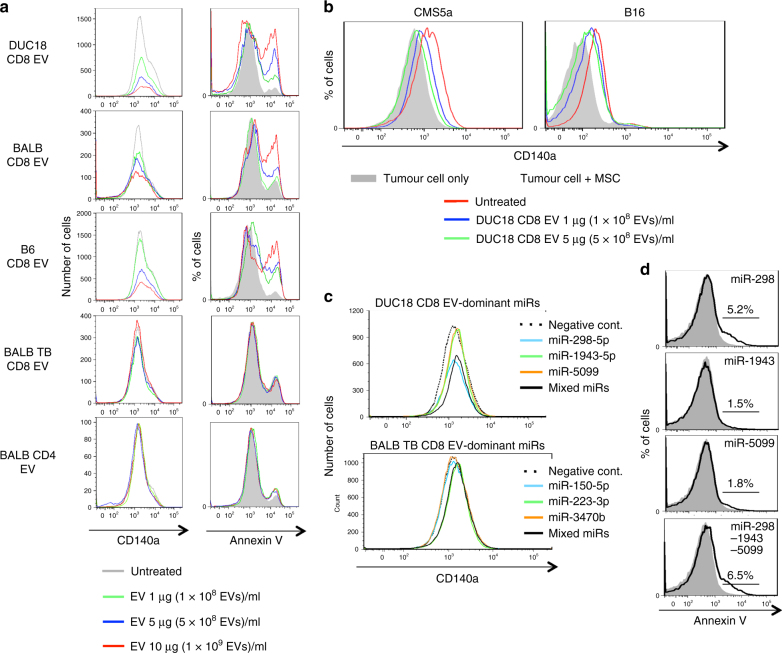
Reduced viability of cultured MSCs by CD8^+^ T cell EVs. **a** DUC18 CD8 EVs, BALB CD8 EVs, B6 CD8 EVs, BALB TB CD8 EVs or BALB CD4 EVs were added to bone-derived MSC cultures at the indicated doses. Three days after EV treatment, bone-derived MSCs (*n* = 5 per group) were stained with anti-CD140a mAb and annexin V and subjected to flow cytometric analysis. **b** CMS5a and B16 cells were cultured with bone-derived MSCs for 4 days in the presence of DUC18 CD8 EVs at the indicated doses (*n* = 4 per group). The obtained tumour cells were analysed for CD140a expression by flow cytometry. **c** Selected miR-298-5p, -1943-3p and -5099 as both DUC18 CD8 EV- and BALB CD8 EV-dominant miRNAs were synthesised and transfected alone or in a mixture by lipofection into bone-derived MSCs (*n* = 4 per group). Synthetic BALB TB CD8 EV-dominant miRNAs (miR-150, 223-3p or 3470b) were used as controls (*n* = 4). 3 days after transfection, the obtained bone-derived MSCs were stained with CD140a- and Sca-1-specific mAbs and annexin V (**d**), and subjected to flow cytometric analysis to assess the total number of cultured MSCs

According to a procedure of camptothecin-induced programmed death of Jurkat cells (Supplementary Fig. 11a)^23^, we examined death of cultured MSCs by addition of CD8^+^ T cell EVs. The number of cultured bone-derived MSCs was markedly decreased with enhanced annexin V-staining intensity at 3 days after treatment with DUC18 CD8 EVs, BALB CD8 EVs and B6 CD8 EVs, but not BALB TB CD8 EVs or BALB CD4 EVs in an activated caspase-3-mediated manner (Fig. 2a; Supplementary Fig. 10b). Furthermore, the generation of mesenchymal-like CMS5a and B16 cells with augmented CD140a expression and spheroid-forming capacity of CMS5a, 4T1, CT26 and B16 cells in the presence of bone-derived MSCs were downregulated in the presence of DUC18 CD8 EVs, but not BALB TB CD8 EVs (Fig. 2b; Supplementary Fig. 11b). These results indicate that progressive tumour cells generated by contact with MSCs were disturbed by CD8^+^ T cell-derived EV-mediated depletion of MSCs.

The cultured MSCs expressed Fas, whereas the cultured CMS5a did not (Supplementary Fig. 12a), suggesting that death molecule ligands, e.g., FasL, tumour necrosis factor (TNF)-α and programmed death (PD)-L1^24,25^, on EV surfaces are involved in the depletion of MSCs. However, FasL, TNF-α or PD-L1 expression was not observed on DUC18 CD8 EVs or BALB CD8 EVs (Supplementary Fig. 12b and d), In addition, a FasL-neutralising mAb did not restore DUC18 CD8 EV-mediated depletion of cultured MSCs (Supplementary Fig. 12c).

After comparison of global-normalised law data from microarray analysis (3D-gene: Toray) among miRNAs from DUC18 CD8 EVs, BALB CD8 EVs, BALB TB CD8 EVs, bone-derived MSCs, CMS5a and B16, we selected miR-298, 1943 and 5099 and miR-150, 223 and 3470b as both DUC18 CD8 EV- and BALB CD8 EV-dominant miRNAs and BALB TB CD8 EV-dominant miRNAs, respectively (Supplementary Table 1). miR-298 and -1943 have been reported to have a suppressive role against tumour progression (Supplementary Fig. 12e). We synthesised six miRNAs for transfection into cultured bone-derived MSCs (Supplementary Fig. 10c). The number of MSCs was reduced by introduction of miR-298 in an activated caspase-3-mediated manner (Supplementary Fig. 10d), but not by introduction of miR-1943 or miR-5099 (Fig. 2c, d). The three BALB TB CD8 EV-dominant miRNAs were not effective (Fig. 2c). In addition, the levels of miR-298-5p in BALB CD8 EVs were confirmed to be higher than those in TB CD8 EVs from B16F10-bearing mice or tumour cell-derived EVs (Supplementary Fig. 10e). These results indicate that CD8^+^ T cells from normal mice, but not tumour-bearing mice, release cytotoxic miRNA (miR-298-5p)-embedded EVs to kill MSCs.

### Dominant EV uptake by mesenchymal tumour stromal cell

**Fig. 3:**
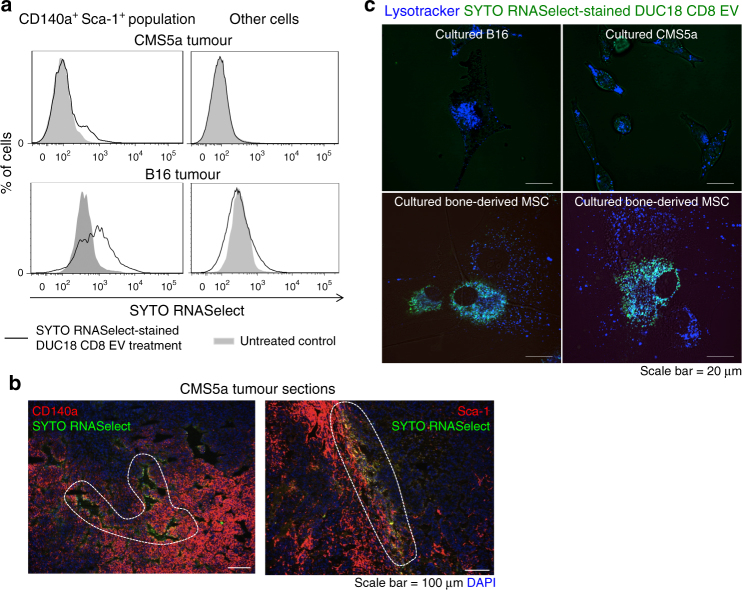
Rapid engulfment of EV RNAs by mesenchymal cells. **a** SYTO RNASelect-stained DUC18 CD8 EVs (10 μg [5–7 × 10^8^ vesicles]/tumour) were injected into CMS5a and B16 tumours (*n* = 3 per group). At 2 h after treatment, the cell suspensions from resected tumours were stained with PE-CD140a- and APC-Sca-1-specific mAbs, and analysed for SYTO RNASelect-derived green fluorescence in the CD140a^+^ Sca-1^+^ population or other cells, including tumour cells, by flow cytometry. **b** SYTO RNASelect-stained DUC18 CD8 EVs (10 μg [5–7 × 10^8^ vesicles]/tumour) were injected into CMS5a tumours (*n* = 3). At 2 h after treatment, the obtained tumours were sectioned and stained with PE-CD140a or PE-Sca-1-specific mAb. Each photo is a representative of 4–5 photos. The dotted circles indicate the overlapping areas of PE-originated red and SYTO RNSelect-derived green fluorescence. **c** SYTO RNASelect-stained DUC18 CD8 EVs (5 μg [4 × 10^8^ vesicles]/mL) were added to in vitro cultured B16, CMS5a or bone-derived MSCs (*n* = 5 per group). Two hours later, the cells were treated with lysotracker (blue), and then observed by confocal laser scanning microscopy. Each photo is a representative of images in 3–4 areas

RNA-specific fluorescence dye (SYTO RNASelect)-stained DUC18 CD8 EVs were introduced into subcutaneous CMS5a or B16 tumours. At 2 h after treatment, green fluorescence of SYTO RNASelect was observed in CD140a^+^ Sca-1^+^ mesenchymal cell populations, including vascular tissues or peritumoral areas, but not in other cell populations including tumour cells (Fig. 3a, b). Unexpectedly, cultured CMS5a and B16 cells could not engulf SYTO RNASelect-stained DUC18 CD8 EVs at 2 h after treatment, whereas endocytic incorporation of SYTO RNASelect-stained EVs was observed immediately in cultured bone-derived MSCs (Fig. 3c). Consistent with the DUC18 CD8 EV findings, SYTO RNASelect-stained BALB CD8 EVs and hPBMC EVs were preferentially engulfed by MSCs compared to B16 or CMS5a tumour cells (Supplementary Fig. 13). Indeed, exosomes appear to have properties that cause preferential engulfment by mesenchymal cells as shown in previous studies^26,27^.

### Prevention of tumour invasion and metastasis by CD8 EV

**Fig. 4:**
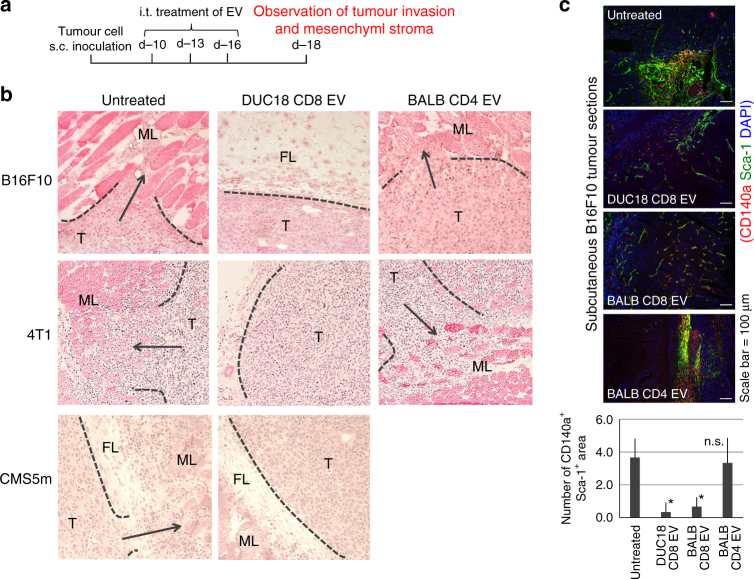
Inhibition of tumour invasion by i.t. treatment of CD8^+^ T cell EVs. **a** Time schedule of the observation of subcutaneous tumour invasion. At days 10, 13 and 16 after subcutaneous B16F10, 4T1 or CMS5m inoculation, DUC18 CD8 EVs, BALB CD8 EVs or BALB CD4 EVs were injected intratumorally (10 μg [5–7 × 10^8^ vesicles]/tumour/day). **b** B16F10, 4T1 or CMS5m tumours on day 18 were sectioned and stained with haematoxylin and eosin (T tumour area, ML muscle layer, FL fatty layer). **c** B16F10 tumours on day 18 were sectioned and stained with PE-CD140a and FITC-Sca-1-specific mAbs and DAPI. Each photo is a representative of 3–4 photos of each group. The number of overlapping areas of PE and FITC fluorescence of DUC18 CD8 EV and BALB CD8 EV-treated groups (*) were significantly different from the untreated group (*n* = 7) with *p* < 0.05. The data were analysed by a two-tailed unpaired Student *t*-test. n.s. not significant

Destruction of the fibroblastic mesenchymal stroma by CD8^+^ T cell-derived EVs may be indirectly associated with the reduced invasive and metastatic properties of progressive tumours^20^. Subcutaneous B16F10, 4T1 or CMS5m tumour was treated with DUC18 CD8 EVs, BALB CD8 EVs, BALB CD4 EVs or BALB TB CD8 EVs at 10, 13 and 16 days after tumour inoculation (Fig. 4a). On day 18, each subcutaneous tumour was carefully removed to observe tumour invasion. In the 50% of untreated, 66.7% of BALB TB EV-treated, and 40% of BALB CD4 EV-treated B16F10 groups, or 50% of untreated and 60% of BALB CD4 EV-treated 4T1 groups, it was impossible to completely remove the tumours and these tumours invaded into the muscle or fatty layer (Fig. 4b; Table 1). Inversely, no invasion of B16F10, 4T1 or CMS5m was observed following treatment with DUC18 CD8 EVs. All remaining mice were able to remove the tumour completely (Table 1). Disappearance of the CD140a^+^ Sca-1^+^ mesenchymal stromal areas in the DUC18 CD8 EV- and BALB CD8 EV-treated groups was observed in day 18 B16F10 sections (Fig. 4c).

**Table 1:** Reduced B16F10 or 4T1 invasion after i.t. treatment of CD8^+^ T cell EVs

I.t. treatment	No. of mice with B16F10 invasion/total mice (%)	No. of mice with 4T1 invasion/total mice (%)
Untreated	6/12 (50%)	2/4 (50%)
DUC18 CD8 EV	0/13 (0%)	1/5 (20%)
BALB TB CD8 EV	2/3 (66.7%)	n.t.
BALB CD8 EV	0/3 (0%)	n.t.
BALB CD4 EV	2/5 (40%)	3/5 (60%)

*n.t.* not tested

Number of B16F10- or 4T1-bearing mice with muscle and/or fatty layer invasion beyond the tumour tissues (impossible to remove the subcutaneous tumours surgically) was counted

**Fig. 5:**
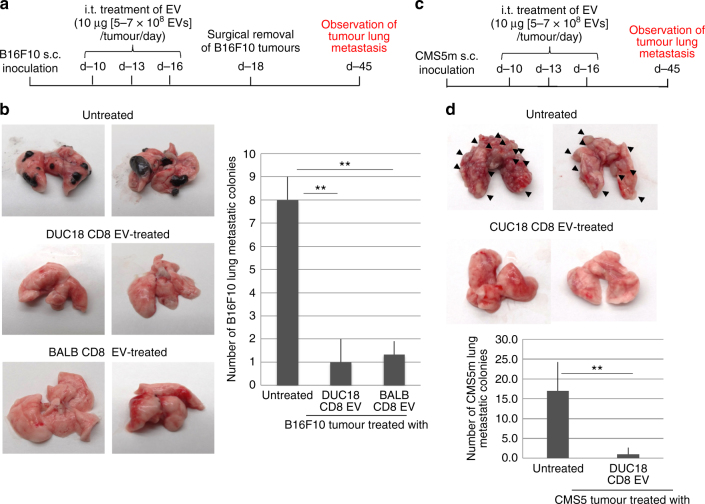
Inhibition of tumour metastasis by i.t. treatment of CD8^+^ T cell EVs. **a** Time schedule of the observation of B16F10 lung metastasis. **b** Lung metastasis of B16F10 tumours on day 45 from the indicated groups (*n* = 7 per group) was examined, and the numbers of metastatic tumour colonies were counted (**p < 0.01; error bars indicate SEM). The data were analysed by two-tailed unpaired Student *t*-test. **c** Time schedule of the observation of CMS5m lung metastasis. **d** Lung metastasis of CMS5m tumours on day 47 (*n* = 6 per group) from the indicated groups was examined, and the numbers of metastatic tumour colonies were counted (**p < 0.01; error bars indicate SEM). The data were analysed by a two-tailed unpaired Student *t*-test

Intratumoural DUC18 CD8 EV and BALB CD8 EV treatments showed a marked decrease in the number of colonies of B16F10 or CMS5m lung metastasis compared to those from untreated, BALB CD4 EV- or CMS5a EV-treated case in wild-type or nude mice studies (Fig. 5; Supplementary Fig. 14a and b). These results highlight the inhibitory roles of CD8^+^ T cell-derived EVs on mesenchymal cell-mediated tumour invasion and metastasis.

### Destruction of tumour stroma by activated CD8^+^ T cell

**Fig. 6:**
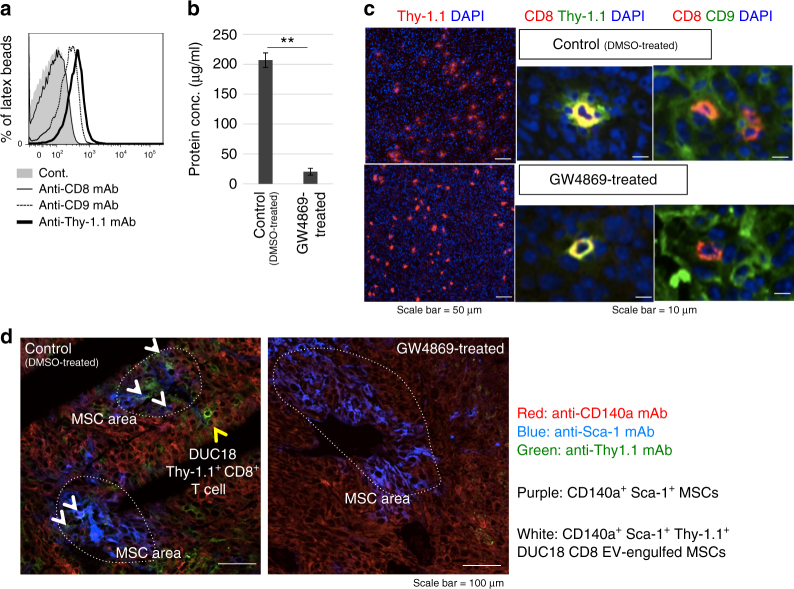
EV production by adoptive transferred tumour-specific CD8^+^ T cells. **a** Cultured Thy-1.1^+^ DUC18 CD8 EV bound to latex beads were stained with the indicated mAbs, and subjected to flow cytometric analysis. **b** EV proteins from the supernatant of Thy-1.1^+^ DUC18 CD8^+^ T cells cultured with/without GW4869 (DMSO treatment as a control) were measured by BCA assay (**p < 0.01; error bars indicate SEM) (*n* = 3). The data were analysed by a two-tailed unpaired Student *t*-test. **c** Thy-1.1^+^ DUC18 CD8^+^ T cells cultured with/without GW4869 were transferred intravenously into CMS5a tumour-bearing BALB/c mice (*n* = 6 per group). The next day, resected CMS5a tumours were sectioned and stained with the indicated FITC-conjugated or PE-conjugated mAbs and DAPI (**c**), or PE-conjugated CD140a-, FITC-conjugated Thy-1.1- and APC-conjugated Sca-1-specific mAbs (**d**). The stained tumour sections were observed by fluorescent microscopy (**c**) or confocal laser scattering microscopy (**d**). Each photo is a representative image from 3–5 areas. The dotted circles and arrows indicate MSC areas (purple) and Thy-1.1^+^ EV-engulfed MSCs (white), respectively

We then investigated whether tumour-infiltrating CD8^+^ T cells can destroy the mesenchymal stromal structure in an EV-mediated manner. Thy-1.1^+^ DUC18 CD8^+^ T cells were cultured with/without GW4869 (an inhibitor of exosome production) and transferred intravenously into CMS5a-bearing mice. Thy-1.1^+^ DUC18 CD8^+^ T cell-derived EVs strongly expressed CD9 and Thy-1.1 and minimally expressed CD8 (Fig. 6a). GW4869-treated Thy-1.1^+^ DUC18 CD8^+^ T cells exhibited lower EV production (Fig. 6b), whereas the degree of infiltration into subcutaneous CMS5a tumours was comparable to the untreated control (DMSO-treated) on the day after intravenous transfer (Fig. 6c, left large photo), indicating that tumour infiltration of CD8^+^ T cells was not altered by GW4869 treatment. The blurred fluorescence of EV-originated Thy-1.1 and CD9 was not visible around tumour-infiltrating GW4869-treated Thy-1.1^+^ DUC18 CD8^+^ T cells (Fig. 6c, right two small photos). The white signals of EV-originated green Thy-1.1 merged with purple CD140a^+^ Sca-1^+^ MSC areas were frequently observed in GW4869-untreated Thy-1.1^+^ DUC18 CD8^+^ T cell-infiltrating CMS5a tumours (Fig. 6d), demonstrating tumour-infiltrating CD8^+^ T cell-derived EV uptake by tumour mesenchymal cells.

**Fig. 7:**
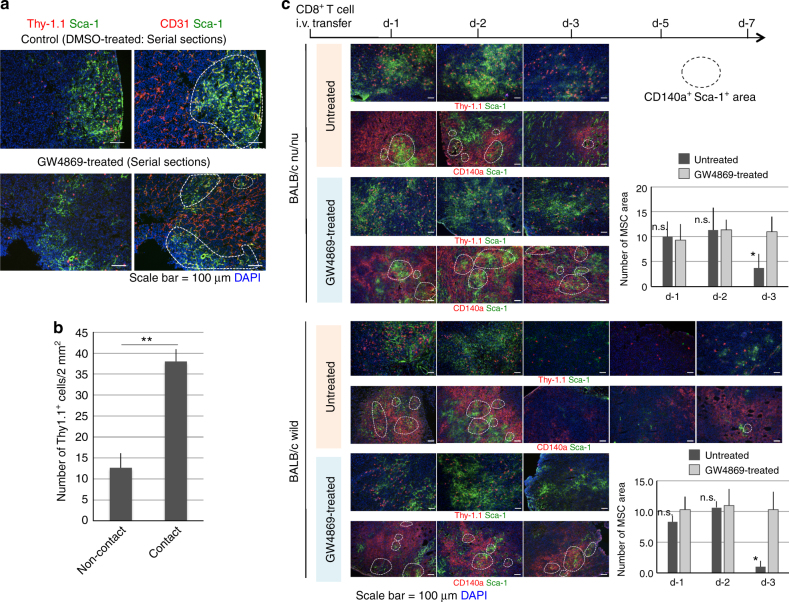
Destruction of mesenchymal tumour stroma by tumour-infiltrating CD8^+^ T cells. **a** DMSO- or GW4869-treated Thy-1.1^+^ DUC18 CD8^+^ T cells were transferred intravenously into CMS5a-bearing BALB/c mice (*n* = 6 per group). The next day, resected tumours were sectioned and stained with the indicated mAbs and DAPI. Each photo is a representative of 4–5 photos. Upper or lower photos of each group are the photos from the serial sections. The dotted lines show the neovascularization areas. **b** Consistent with **a**, CMS5a sections of DMSO-treated Thy-1.1^+^ DUC18 CD8^+^ T cell-transferred groups were stained with PE-conjugated Thy-1.1-specific mAb and FITC-conjugated Sca-1-specific mAb, and then the number of Thy-1.1^+^ cells in contact with/without Sca-1^+^ cells was counted (**p < 0.01; error bars indicate SEM). The data were analysed by a two-tailed unpaired Student *t*-test. **c** At days 1, 2, 3, 5 and 7 after transferring untreated or GW4869-treated Thy 1.1^+^ DUC18 CD8^+^ T cells into CMS5a-bearing BALB/c wild-type or nude mice (*n* = 5 per group), the resected tumours were sectioned and stained with the indicated mAbs and DAPI. Each photo is a representative of 3–4 photos. The dotted circles show MSC areas overlapping with PE fluorescence of CD140a with FITC fluorescence of Sca-1. The number of overlapping areas of the untreated group on day 3 was significantly different from that in the GW4869-treated group in BALB/c nu/nu- and BALB/c wild-type cases (*p < 0.05; n.s. not significant; error bars indicate SEM). The data were analysed by a two-tailed unpaired Student *t*-test

Interestingly, DUC18 CD8^+^ T cells, regardless of GW4869 treatment, entered CMS5a tumour lesions through the Sca-1^+^ CD31^+^ VCAM-1^+^ neovascular areas, where stromal endothelial progenitor cells (CD31^+^ Sca-1^+^) and MSCs (CD140a^+^ Sca-1^+^) were abundantly observed (Fig. 7a, b; Supplementary Fig. 15a–c) on the day after intravenous treatment. CD140a^+^ Sca-1^+^ mesenchymal stromal areas of CMS5a tumours in BALB/c wild-type or nude mice disappeared on day 3 after transfer of GW4869-untreated Thy-1.1^+^ DUC18 CD8^+^ T cells (Fig. 7c) and persisted thereafter (Fig. 7c: in BALB/c wild-type), demonstrating a correlation between tumour infiltration of EV-producing CD8^+^ T cells and depletion of the mesenchymal stroma.

## Discussion

CD8^+^ T cell-derived EV-mediated depletion of mesenchymal tumour stromal cells including MSCs and CAFs leads to vigorous attenuation of tumour progression as shown by the loss of invasive and metastatic properties (Supplementary Fig. 16). Recently, it has been reported that tumour stem cells characterised as mesenchymal-like invasive tumour cells can be reprogrammed into an epithelial state by cyclic (c) AMP-mediated activation of protein kinase A (PKA) and subsequent epigenetic promotion of epithelial-related genes by histone demethylase^28^. Consequently, destruction of mesenchymal tumour stroma by CD8^+^ T cell EVs may elicit mesenchymal-to-epithelial transition of invasive tumours in a cAMP/PKA-mediated manner, but it remains unclear whether unknown actions, including reduction of TGF-β expression, participate in this tumour reprogramming. In contrast, we found that the culture period and biological conditions are pivotal factors in obtaining functional EVs. It was previously reported that the quality and quantity of antigen-presenting efficacy of DC-derived EVs is inversely correlated with the maturation process of DCs in a culture system^29,30^. Thus, it is necessary to explore a wide variety of culture conditions to obtain functional immune cell-derived EVs. Our findings indicate that targeting of certain EVs from CD8^+^ T cells to mesenchymal tumour stromal cells is useful for future treatment of patients with metastatic refractory tumours, including pancreatic cancer.

In addition to the central effect against malignancy by specific interactions with tumour cells^17,31,32^, we demonstrated that CD8^+^ T cells are involved in restoration of the tumour microenvironment by EV-mediated regulation of mesenchymal tumour stroma. miR-298–5p in CD8^+^ T cell EVs is one of the molecules involved in the depletion of tumoural mesenchymal cell populations, although it is necessary to elucidate the mechanism of action including identification of target genes. In addition to EV-embedded cytotoxic miRNA, death molecule ligands such as FasL, TNF-α and PD-L1 on CD8^+^ T cell EVs are candidates for depletion of tumour stromal mesenchymal cells. However, these molecules are not expressed on CD8^+^ T cell EVs. Future studies should examine the involvement of cytotoxic molecules, e.g., granzyme B, in CD8^+^ T cell EVs as indicated in Supplementary Fig. 2 and reported by other groups in studies of NK cell EVs^33,34^.

Circulating MSCs are primarily retained near tumour neovascularization and angiogenesis sites as pericytes to protect blood walls^35^. In addition, the differentiation of tumour-infiltrating MSCs to myofibroblasts is promoted by neovasculature development-related cytokines^5,36^. Thus, formation of the mesenchymal tumour stromal structure is strongly associated with the angioarchitecture. As observed in (Fig. 7b), activated tumour-specific CD8^+^ T cell infiltration into the tumour stromal areas with neovascularization in which epithelial progenitor cells (EPCs) concomitantly accumulate from the periphery^37^ is morphologically convenient for EV-mediated elimination of mesenchymal tumour stroma. GW4869 is known to attenuate production of inflammatory cytokines such as interleukin (IL)-1β, IL-6 and TNF-α of macrophages^38^. Therefore, GW4869-treated CD8^+^ T cells that reduced cytokine production may regulate tumour angiogenesis without affecting their tumour infiltration.

In addition to the abnormalities of tumour blood vessels and interstitial pressure towards the vessel lumen, reduced expression of adhesion molecules for rolling and firm adhesion of lymphocytes on tumoural endothelial cells (e.g., intercellular adhesion molecule-1 and -2 for lymphocyte function-associated antigen-1, and vascular adhesion molecules [VCAM]-1 for very late antigen [VLA]-4) is the cause of impaired extravasation of lymphocytes into tumour lesions^39–42^. Down-regulation of adhesion molecules on endothelial cells is induced by chronic stimulation with angiogenic factors such as basic fibroblast growth factor (bFGF) and vascular endothelial growth factor (VEGF)^43^. Our results regarding infiltration of transferred CD8^+^ T cells at the tumour neovascularization sites show the successful extravasation of the transferred CD8^+^ T cells via new tumour blood vessels where endothelial cells still bear adhesion molecules because of a lack of continuous stimulation with bFGF and VEGF. A previous study demonstrated the necessity of EPC activation via VCAM-1 signalling in the extravasation of VLA-4-expressing lymphocytes^44^.

## Methods

### Mice and tumour cell lines

Female B6 and BALB/c mice were purchased from Japan SLC. Congenic BALB/c Thy-1.1 mice with mutated ERK2 (mERK2) 136-144 (QYIHSANVL)-specific H-2K^d^-restricted TCR (Vα10.1/Jα48 and Vβ8.3/Dβ2.1/Jβ2.6) gene-transfected DUC18 mice, and Congenic DUC18 Thy-1.1 mice were bred and maintained in a specific pathogen-free vivarium at Mie University Institute of Laboratory Animals. All mice were used at 7–9 weeks of age. In all experiments, tumour-bearing mice were randomly allocated to experimental groups. In experiments where in vivo assays were performed, the variation within groups allowed the detection of differences with 4–5 mice per group. CMS5a, CMS5m, CMS7, CT26, 4T1, B16 and B16F10 tumour cell lines were maintained in D-MEM medium supplemented with 10% foetal calf serum (FCS). Jurkat E6-1 cell line was cultured in RPMI-1640 medium supplemented with 10% FCS. All cell lines were purchased from ATCC and were found to be negative for mycoplasma contamination. Experimental protocols were approved by the Animal Ethics Committee of Mie University, Tsu, Japan (Approval number 23–8). The human study was conducted in accordance with the current version of the Declaration of Helsinki. Written informed consent was obtained from all healthy donors participating in this study. The experimental protocols were approved by the Ethics Review Committees of the Mie University, Tsu, Japan (Approval No.: 2879).

### Preparation of EVs from culture media

EV-free FCS (dFCS) was prepared by centrifugation of FCS at 100,000 × *g* for 18 h followed by filtration (0.45 and 0.22 nm). Splenocytes (2 × 10^7^ cells/mL) from DUC18 and Thy-1.1 DUC18 mice were cultured in RPMI-1640 supplemented with 10% dFCS and 1 µg/mL mERK2 peptides. Splenocytes (2 × 10^7^ cells/mL) from B6 mice were cultured in RPMI-1640 supplemented with 10% dFCS and 1 µg/mL each of TRP-2 (SVYDFFVWL) and gp100 (EGSRNQDWL) peptides^45^. Splenocytes (2 × 10^7^ cells/mL) from BALB/c, 2 weeks subcutaneous CMS5a- or B16F10-bearing BALB/c, or CD8^+^ T cell-depleted BALB/c mice were cultured with RPMI-1640 supplemented with 10% dFCS and 1 µg/mL anti-CD28 mAb (37.51: eBioscience) in anti-CD3 mAb (2C11: 2 µg/mL: Biolegend)-immobilised 12-well plates. CD8^+^ hPBMCs separated negatively with hCD4-beads (Miltenyi Biotec) were cultured with RPMI-1640 supplemented with 10% dFCS and 1 µg/mL CD28.2 mAb (Biolegend) in OKT3 mAb (2 µg/mL: Biolegend)-immobilised 12-well plates. CD8^+^ T cell-depleted BALB/c mice were prepared by intravenous injection of a Lyt-2.2-specific mAb (400 μg/mouse) to expand CD4^+^ T cells in vitro. After 4 days of cultivation, each culture medium was changed to RPMI-1640 supplemented with 10% dFCS and recombinant (r) IL-2 (100 IU/mL) for 3 days. The obtained supernatants were used as EV sources. The cultured cells were subjected to flow cytometric analysis using murine anti-CD4 (GK1.5), CD8 (53–6.7), TCRVβ (H57–597), TCRVβ8.3 (1B3.3), CD9 (MZ3), CCR7 (4B12), FasL (Kay-10), PD-1 (RMP1-30), CD40L (MR1) or TIM-3 (RMT3–23) mAb, or human CD4 (OKT4), CD8 (RPA-T8) and TCRαβ (IP26)-specific mAbs (all from Biolegend).

The obtained culture supernatants (approximately 500 mL) were first centrifuged at 10,000×*g* for 20 min, filtrated through 0.45- and 0.22-μm filters, and subjected to ultrafiltration to concentrate the sample to 100 mL (Kvick Lab Packet 50 KD: GE Healthcare). The concentrated culture supernatants were further filtrated through a 0.22-μm filter and subjected to ultracentrifugation for 2 h at 100,000×*g* (SW28 rotor: Beckman Coulter). The obtained EV pellets were suspended in PBS, recentrifuged at 100,000×*g*, dissolved in 0.5–2 mL PBS, and stored at 4 °C.

The protein concentrations of the purified EVs and parent cells were assessed using the bicinchoninic acid (BCA) protein assay kit (Pierce). The mean number and diameter of purified EVs were measured by using a nano-tracking system (LM10-HS: NanoSight). EV surface proteins were detected by flow cytometric analysis of the latex beads-bound EVs stained with fluorescein isothiocyanate (FITC) or phycoerythrin (PE)-conjugated anti-CD4, CD8, CD9, CD63 (NVG-2), Vβ8.3 (1B3.3), CCR7, FasL, PD-1, CD40L or TIM-3 mAb (all from Biolegend). Polystyrene latex beads (10 μm diameter) were mixed with EV solutions at a ratio of 3 particles/latex bead in 0.1 M 2-morpholinoethanesulfonic acid buffer, incubated for 2 h on a rotating shaker, and then blocked with 400 mM glycine. The obtained latex bead-bound EVs were washed twice with PBS containing 2% dFCS and subjected to staining with mAbs.

To investigate the kinetics of EVs in vivo and in vitro, 100–300 µg of EVs were incubated with 10 µM SYTO RNASelect (Molecular Probes) at 37 °C for 20 min, and then subjected to a Spehadex-G25 spin column to exclude free dye.

### Preparation of bone-derived MSCs

Bone-derived MSCs were prepared from bone-marrow-cell flushed thighbones (femurs) according to the manufacturer’s directions (StemCell Technologies). Both ends of 10 femurs from BALB/c or Thy 1.1^+^ BALB/c mice were cut down and transferred into a mortar in 5 mL of PBS containing 1% bovine serum albumin (BSA) and crushed by gentle stirring with a pestle for 5 min to remove as many red bone marrow cells as possible. After 5 cycles of removing red bone marrow cells, the fragmented white femurs were collected and incubated with PBS supplemented with 0.2% collagenase type I (Sigma). After vigorous shaking at 37 °C for 40 min in a water bath, the supernatants containing MSCs were washed three times with PBS. Dish-adherent MSCs were cultured for 30 days in mouse MesenCult MSC basal medium supplemented with 20% MSC stimulatory supplement (StemCell Technology), with the medium changed every other day. The potential of the obtained MSCs to differentiate into adipocytes and osteocytes was tested by using 70% confluent MSCs in MesenCult MSC basal medium supplemented with 20% adipogenic and osteogenic stimulatory supplements for 2 weeks, and then staining with Oil Red O (Sigma-Aldrich) for adipocytes and Alizarin Red S (Wako Pure Chemical Industries) for osteocytes. Colony formation in primary MSCs cultivated in MSC stimulatory supplement-containing medium was observed after staining with Giemsa (Wako Pure Chemical Industries). The purity of cultured bone-derived MSCs was confirmed to be over 95% by flow cytometric analysis (FACScant II: BD Biosciences) using both PE-conjugated anti-CD140a and FITC-conjugated anti-Sca-1 mAbs. For more precise analysis of the cultured MSCs, the positivity of CD29 and CD105 and negativity of CD14, CD19, CD34 and CD45 of MSCs were analysed by flow cytometry using each molecule-specific mAbs (all from Biolegend).

### Preparation of bone-derived MSC-chimeric mice

Bone marrow cells were prepared by marrow flushing of the femurs of BALB/c mice (Thy-1.2) as described above. BALB/c mice were irradiated at 6 Gy before cultured MSC transplantation. The cultured bone-derived MSCs from Thy-1.1^+^ BALB/c mice (1 × 10^6^) were mixed with 5 × 10^6^ cells of bone marrow cells from BALB/c femurs, transferred intravenously into irradiated BALB/c mice, and the resulting chimeric mice were maintained with autoclaved water containing 1 mg/mL neomycin sulphate (Calbiochem) and X-ray-sterilised food for over 2 weeks. At 60 days after MSC transfer, Thy-1.1^+^ bone-derived MSC-chimeric BALB/c mice were used to confirm tumour MSCs.

### Treatment with CD8^+^ T cells and EVs in vivo

10 days after tumour inoculation (approximately 10 mm tumour diameter), CMS5a-bearing BALB/c or BALB/c nude mice were infused intravenously with 7-day cultured Thy-1.1^+^ DUC18 CD8^+^ T cells (1 × 10^7^ cells per mouse) with/without GW4869 treatment simultaneously with intratumoral treatment of 1 µg per tumour anti-murine glucocorticoid-induced TNF receptor-related protein mAb (DTA-1). DTA-1 was used as an accelerator of CD8^+^ T cell accumulation at tumour sites^46^. GW4869 treatment was performed at 20 μg/mL for 24 h before the end of culture. At 1, 2, 3, 5 and 7 days after infusion, CMS5a tumour tissues were collected and subjected to fluorescence immunohistochemistry.

CMS5a, CT26 or B16 cells (1 × 10^6^ cells per mouse) were inoculated subcutaneously in the back skin of BALB/c or B6 mice, respectively. At 12 days after the randomised and blinded allocation of all tumour-bearing mice, EVs were treated intratumorally (10 μg [5–7 × 10^8^ vesicles]/tumour), and subsequent tumour diameter was measured. Alternatively, 3 days after EV treatment, the excised tumours were cut out with scissors and then incubated in PBS containing 0.5% trypsin and 1 mM EDTA at 37 °C for 60 min. The obtained tumour cell suspensions were passed through wool columns, washed three times with PBS containing 1% FCS, and then subjected to flow cytometric analysis using FITC-conjugated mAbs specific for Sca-1, I-A^d^, CD11b CD11c, CD73 or CD206 and PE-conjugated mAb specific for F4/80, Gr-1 or CD140a (all from Biolegend), or cultivation with RPMI-1640 supplemented with 10% FCS (1 × 10^5^ cells/mL) to evaluate spheroid formation.

EVs (10 μg [5–7 × 10^8^ vesicles]/tumour) were injected i.t. in CMS5a-bearing Thy-1.1^+^ bone-derived MSC-chimeric BALB/c mice 2 weeks after subcutaneous CMS5a inoculation. The suspensions of tumour cells obtained 3 days after EV treatment were stained with FITC-conjugated Thy-1.1-specific mAb, PE-conjugated CD140a-specific mAb, and/or allophycocyanin (APC)-conjugated Sca-1-specific mAb and subjected to flow cytometric analysis after exclusion of 7-aminoactinomycin D (all from Biolegend)-stained cells.

DUC18 CD8 EVs, BALB CD8 EVs, BALB CD4 EVs or CMS5a EVs (10 μg [5–7 × 10^8^ vesicles]/tumour/day) was injected in d-10, d-13 and d-16 subcutaneous B16F10 on B6 mice, 4T1 on BALB/c mice, or CMS5m on BALB/c wild-type or nude mice. At 18 days after tumour inoculation, grown B16F10 tumours (~2 cm in diameter) were carefully excised in the B16F10-using case with scissors and the skin was closed with surgical sutures. At 45 days after tumour inoculation, lung metastasis of each tumour was observed.

### Treatment with CD8^+^ T cell-derived EVs in vitro

DUC18 CD8 EVs, BALB TB CD8 EVs, B6 CD8 EVs, BALB CD8 EVs or hPBMC EVs were added at 5 μg [2–4 × 10^8^ vesicles]/mL in 5 × 10^4^ cells/mL CMS5a, B16 or bone-derived MSC culture, or a mixed culture of 5 × 10^4^ cells/mL CMS5a or B16 cells together with 5 × 10^4^ cells/mL of bone-derived MSCs for 3 days at 1, 5 or 10 µg (1, 4 or 8 × 10^8^ EVs)/mL. At 3 days after cultivation, the obtained cells were subjected to observation of spheroid formation and flow cytometric analysis for counting total cell numbers and the expression of CD140a and/or Sca-1, or annexin V, according to analysis of 5 μM camptothecin (Wako Pure Chemical Industries)-induced death of Jurkat cells for 5 h.

### Fluorescent immunohistochemistry

Frozen CMS5a and B16F10 tumour specimens embedded in O.C.T compound (Sakura Finetech) were sectioned at a thickness of 3 μm, air-dried for 2 h, fixed with ice-cold acetone for 15 min, and subjected to immunohistochemistry. After washing three times with PBS, the tissue slides were incubated at 4 °C in blocking solution (PBS supplemented with 1% BSA, 5% Blocking One Histo [Nacalai Tesque]) and 0.2 µg/mL anti-mouse CD16/CD32 mAb (Biolegend) for 30 min. The tumour sections on the slides were then dual-labelled with PE-conjugated and FITC-conjugated mAbs, or triple-stained with PE-conjugated CD140a, APC-conjugated Sca-1 and FITC-conjugated Thy-1.1-specific mAbs diluted with PBS supplemented with 1% BSA and 5% Blocking One Histo for 1 h at room temperature in a humidified chamber. After washing three times with PBS supplemented with 0.02% Tween-20, the slides were mounted in Prolong Gold antifade reagent with/without DAPI (Life Technologies), and observed by fluorescence microscopy (BX53F; Olympus Co. Ltd.; Tokyo, Japan) or confocal laser scanning microscopy (LSM780; Carl Zeiss, Oberkochen, Germany). The photographs from PE-, FITC- and DAPI- or PE-, APC- and FITC-stained tissue sections were merged by Photoshop elements software (Adobe Systems Software).

### Observation of EV uptake in vivo and in vitro

DUC18 CD8 EVs were stained with SYTO RNASelect (Life Technologies) and purified with PD SpinTrap G25 (GE Healthcare) as described above. SYTO RNASelect-stained EVs were injected into day-14 subcutaneous CMS5a or B16 tumours at 20 μg per tumour. At 2 h after injection, tumours were resected and suspended in 0.25% trypsin/0.02% EDTA. After washing three times with PBS/10% FCS, the obtained tumour cell suspension was stained with PE-conjugated anti-CD140a mAb and APC-conjugated anti-Sca-1 mAb (Biolegend), followed by flow cytometric analysis.

CMS5a cells, B16 cells or bone-derived MSCs were cultured in four compartments (Greiner Bio One) at an initial density of 1.0 × 10^4^ cells/well overnight. The next day, the SYTO RNASelect-labelled DUC18 CD8 EVs, BALB CD8 EVs or hPBMC EVs were added to the cell cultures at a concentration of 5 μg [2–4 × 10^8^ vesicles]/mL. After 2 h, the cells were stained by LysoTracker Blue (Life Technologies) and observed by confocal laser scanning microscopy (LSM780; Carl Zeiss) equipped with 405-nm diode laser (for LysoTracker Blue) and 488-nm argon laser (for SYTO RNASelect).

### Analysis of EV miRNAs

EVs were dissolved with TRIzol reagent (Thermo Fisher Scientific) and total miRNAs were purified using the miRNeasy mini kit (Qiagen). Twenty µg of EVs were analysed with a 3D-gene miRNA microarray system (Toray). According to the RNA sequences from miRBase, selected DUC18 CD8 EV-dominant miR-298–5p, 1943-5p and 5099; and BALB TB CD8 EV-dominant miR-150-5p, 223-3p and 3470b were synthesised (Hokkaido System Science).

To study of activated caspase-3-mediated apoptosis, pan-caspase inhibitor (Z-VAD-FMK: MedChem Express) or caspase-3 inhibitor (Z-DEVD-FMK: BD Biosciences) was added to the MSC culture at 20 μM with 10 μg DUC18 CD8 EVs. At 2 days after treatment, MSCs were stained with APC-conjugated annexin V and analysed by flow cytometry.

Small RNAs were isolated from EVs using miRNeasy mini kit (Qiagen) according to the manufacturer’s directions. Reverse transcription of RNAs was performed using the Mir-X miRNA First-Strand Synthesis Kit (Clonetech). RT-qPCR was performed using the StepOnePlus Real-Time PCR system (Applied Biosystems) with SYBR Advantage qPCR Premix (Clonetech) and synthetic primers (GeneDesign: miR-298–5p; GGCAGAGGAGGGCTGTTCTTCCC). The quantity of each miRNA was measured by the comparative Ct method (the ^⊿⊿^Ct method). The level of each miRNA was normalised to that of a U6 snRNA control.

### Western blot analysis

Cell proteins were extracted with cell lysis buffer (Cell Signaling Technology) according to the manufacturer’s directions. EV and cell proteins were dissolved in Laemmli sample buffer (Bio-Rad) with (for perforin, granzyme B, Alix and Tsg101 detection) or without (for CD9 detection) 5% 2-ME, and boiled for 5 min. Five μg of each protein sample and molecular weight markers (MagicMark XP Standard: Thermo Fisher Scientific) were separated by 10% polyacrylamide gel electrophoresis (e-PAGEL E-R10L: ATTO Corporation) using SDS running buffer (Bio-Rad) at 40 mA for 60 min. The resulting gel was soaked three times in transfer buffer (Bio-Rad) and proteins were transferred to an Immobilon-P polyvinylidene fluoride membrane (Merck) with a Trans-Blot SD Semi-Dry Transfer Cell (Bio-Rad) at 1 mA/cm^2^ for 1 h. The obtained membrane was blocked with 5% skim milk (Wako Pure Chemical Industries) in 0.05% TTBS (TBS containing 0.05% Tween-20), and then treated with a primary mAb specific for CD9 (eBioKMC8: eBioscience), perforin (eBioOMAK-D: Thermo Fisher Scientific), granzyme B (21631: R&D Systems), Alix (3A9: Biolegend) or Tsg101 (EPR7130: Abcam) at 4 °C overnight. After washing three times with 0.05% TTBS, the membrane was incubated with horseradish peroxidase-conjugated secondary Ab specific for mouse IgG (GE Healthcare), rat IgG (R&D Systems) or rabbit IgG (MBL International Corporation) at room temperature for 1 h. After washing four times with 0.05% TTBS, the membranes were treated with ECL plus (GE Healthcare) and visualised with LAS-4000 (Fujifilm).

### Statistical analysis

Data comparison between two groups were evaluated by the analysis of two-tailed unpaired Student *t*-test. *p* values below 0.05 were considered statistically significant.

### Data availability

The authors declare that all data supporting the findings of this study are available within the article and its supplementary information files or from the corresponding authors on reasonable request.

## Electronic supplementary material

Supplementary Information
