# Supplementary material for: Activated CD8+ T cell extracellular vesicles prevent tumour progression by targeting of lesional mesenchymal cells
Source: Nat Commun. 2018 Jan 30;9:435. doi: 10.1038/s41467-018-02865-1 (PMC5789986; doi:10.1038/s41467-018-02865-1)
Supplement: Supplementary file 1 — Supplementary Information [file 41467_2018_2865_MOESM1_ESM.pdf]

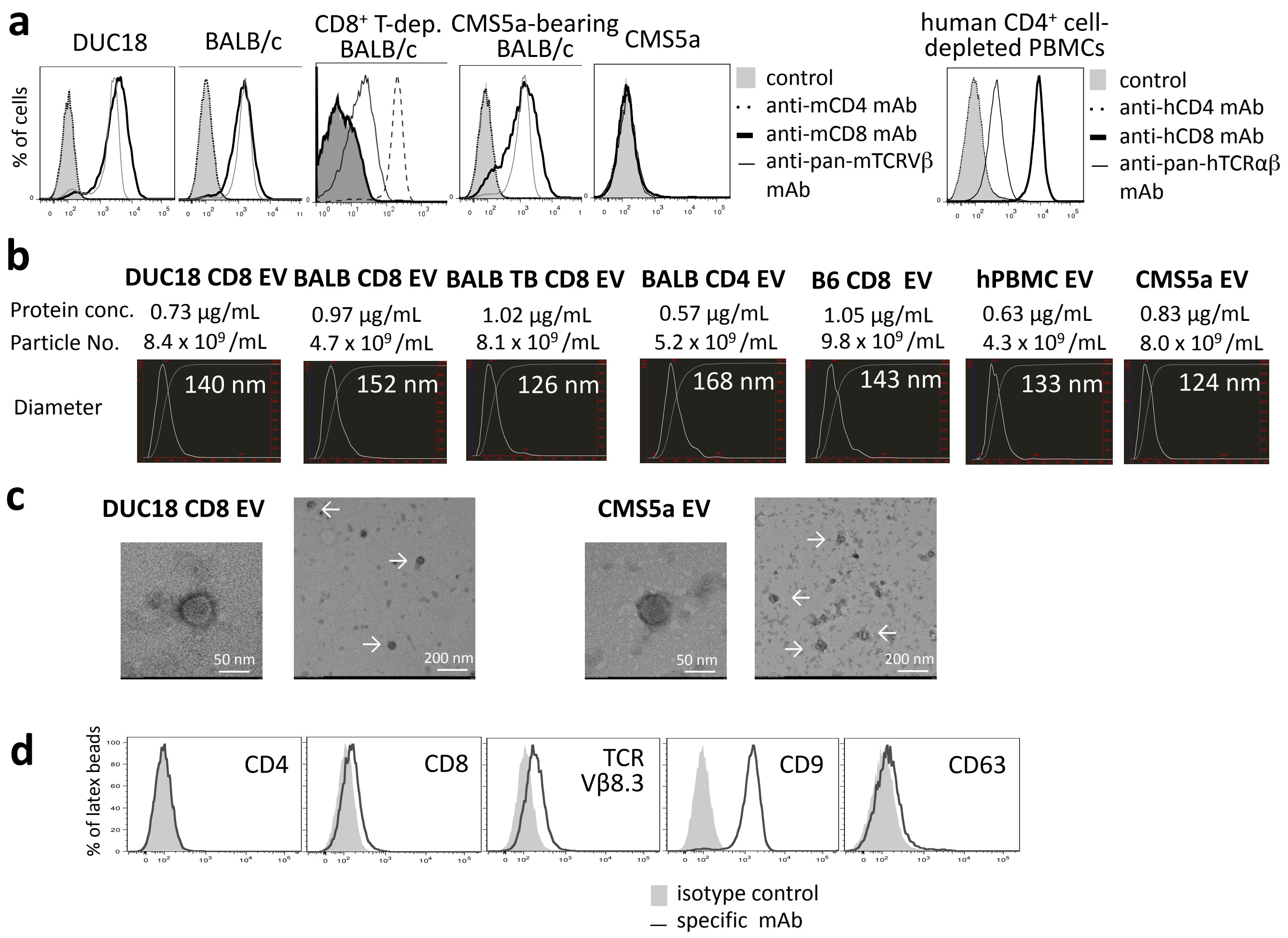

Supplementary Figure 1. Characterization of donor cells and derived EVs. (a) DUC18, BALB/c, CD8$^+$ T cell-depleted BALB/c, and CMS5a-bearing BALB/c splenocytes, CMS5a, and CD4$^+$ cell-depleted hPBMCs cultured for 7 days were subjected to flow cytometric analysis using the indicated mAbs. (b) DUC18 CD8 EVs, BALB CD8 EVs, BALB TB CD8 EVs, BALB CD4 EVs, B6 CD8 EVs, hPBMC EVs, and CMS5a EVs were measured to determine protein concentration by the BCA assay, and total particle numbers and mean diameters were determined by nano-tracking analysis. (c) DUC18 CD8 EVs and BALB TB CD8 EVs were observed by transmission electron microscopy at low and high magnifications. (d) Latex beads bound DUC18 CD8 EVs were stained with the indicated mAbs and examined by flow cytometry. All EVs were obtained at 0.6-1.0 μg/mL protein concentrations and approximately 4-10 x 10^9 numbers/mL, and were confirmed to have 110-150 nm mean diameters. DUC18 CD8 EVs were shown to have modest expression of CD8, CD63, and TCRVβ8.3, with substantial expression of CD9.

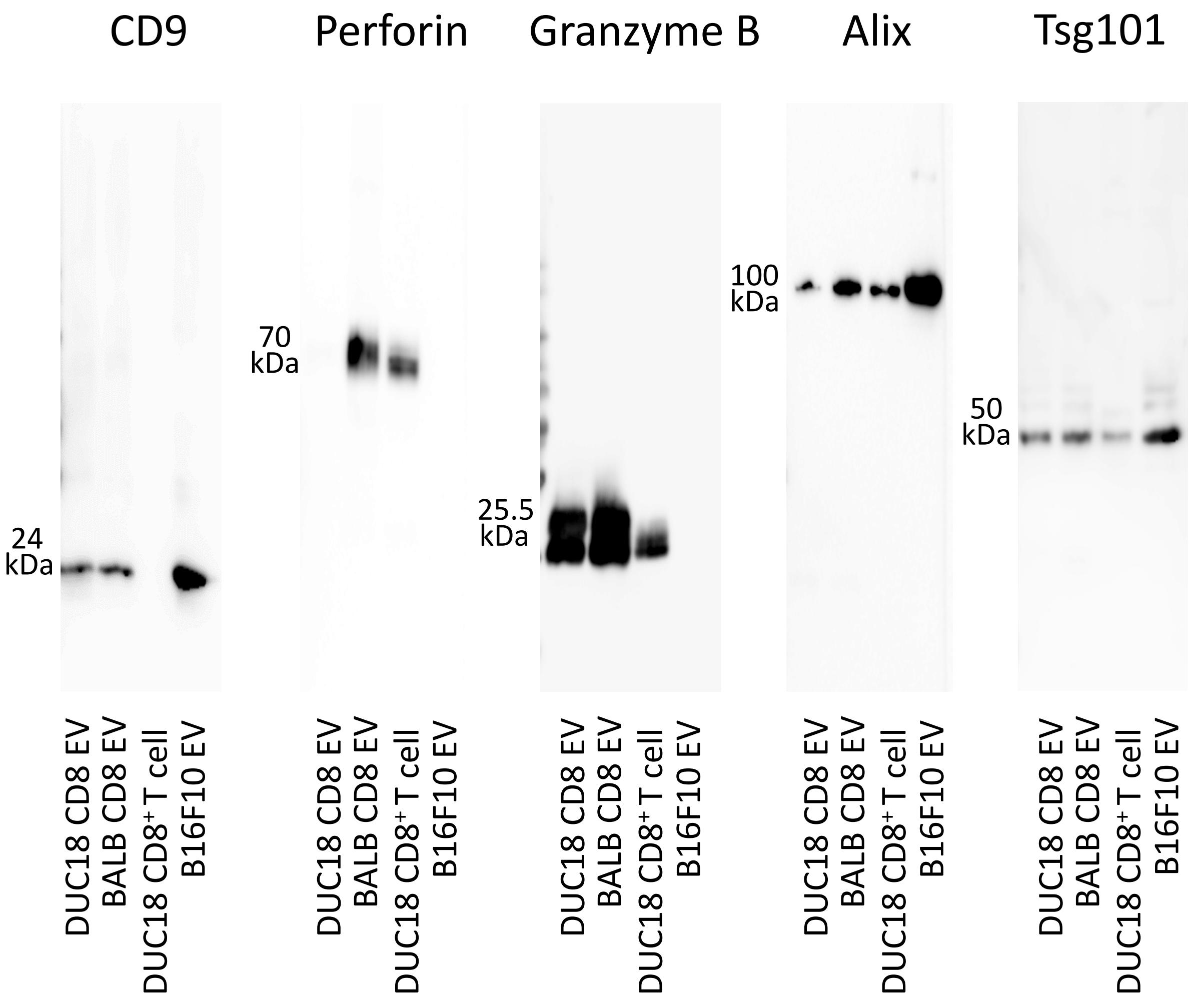

Supplementary Figure 2. Western blot analysis of EV proteins. Total proteins from DUC18 CD8 EVs, BALB CD8 EVs, B16F10 EVs, or DUC18 CD8^{+} T cells were subjected to SDS-PAGE, and western blot analysis was performed by using mAbs specific for CD9, perforin, granzyme B, Alix, and Tsg101.

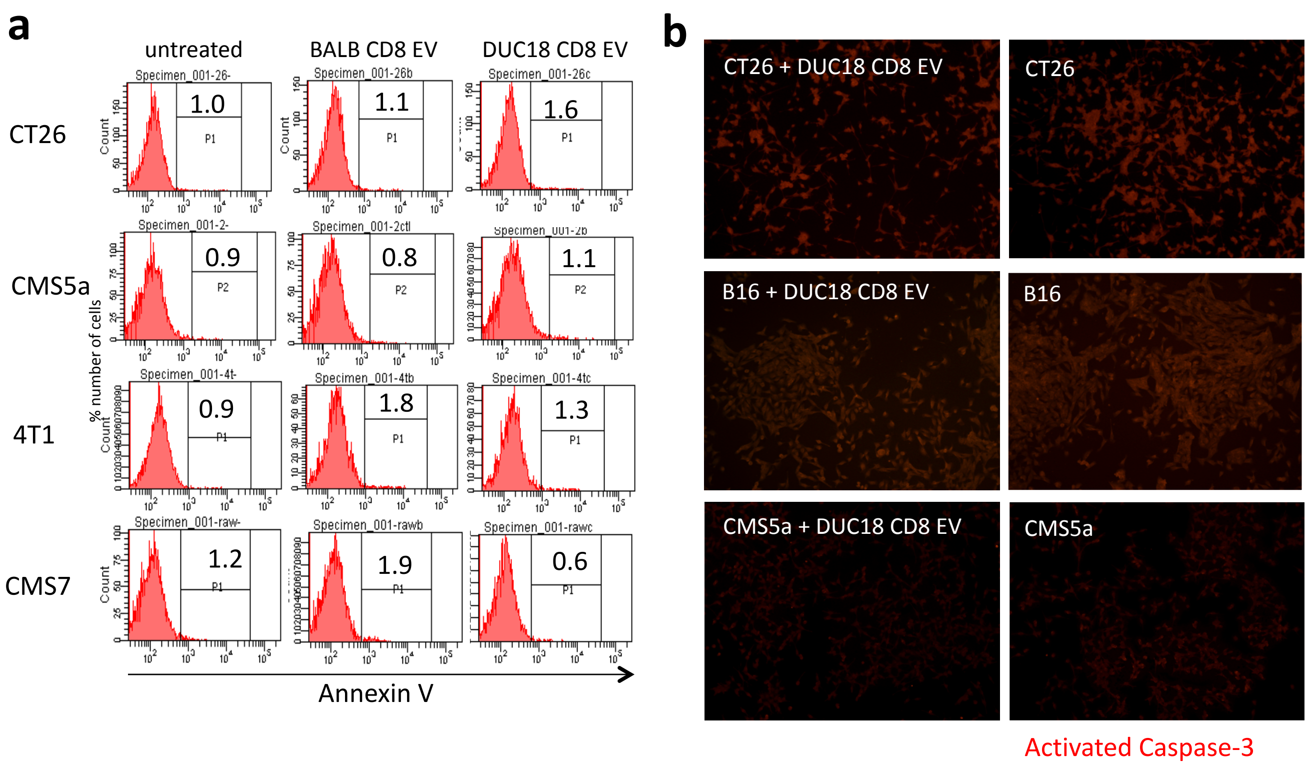

Supplementary Figure 3. No effect of CD8$^+$ T cell EVs on cultured tumour cells. (a) DUC18 CD8 EVs or BALB CD8 EVs were added to the cultures of CT26, CMS5a, 4T1, and CMS7 (10 μg/mL). Three days after EV treatment, the tumour cells were stained with annexin V and then analysed by flow cytometry. (b) CT26, B16 or CMS5a cultured with/without DUC18 CD8 EVs for 2 days were fixed and permeabilized with acetone, and then stained with an activated caspase-3-specific mAb (C92-605: BD Biosciences).

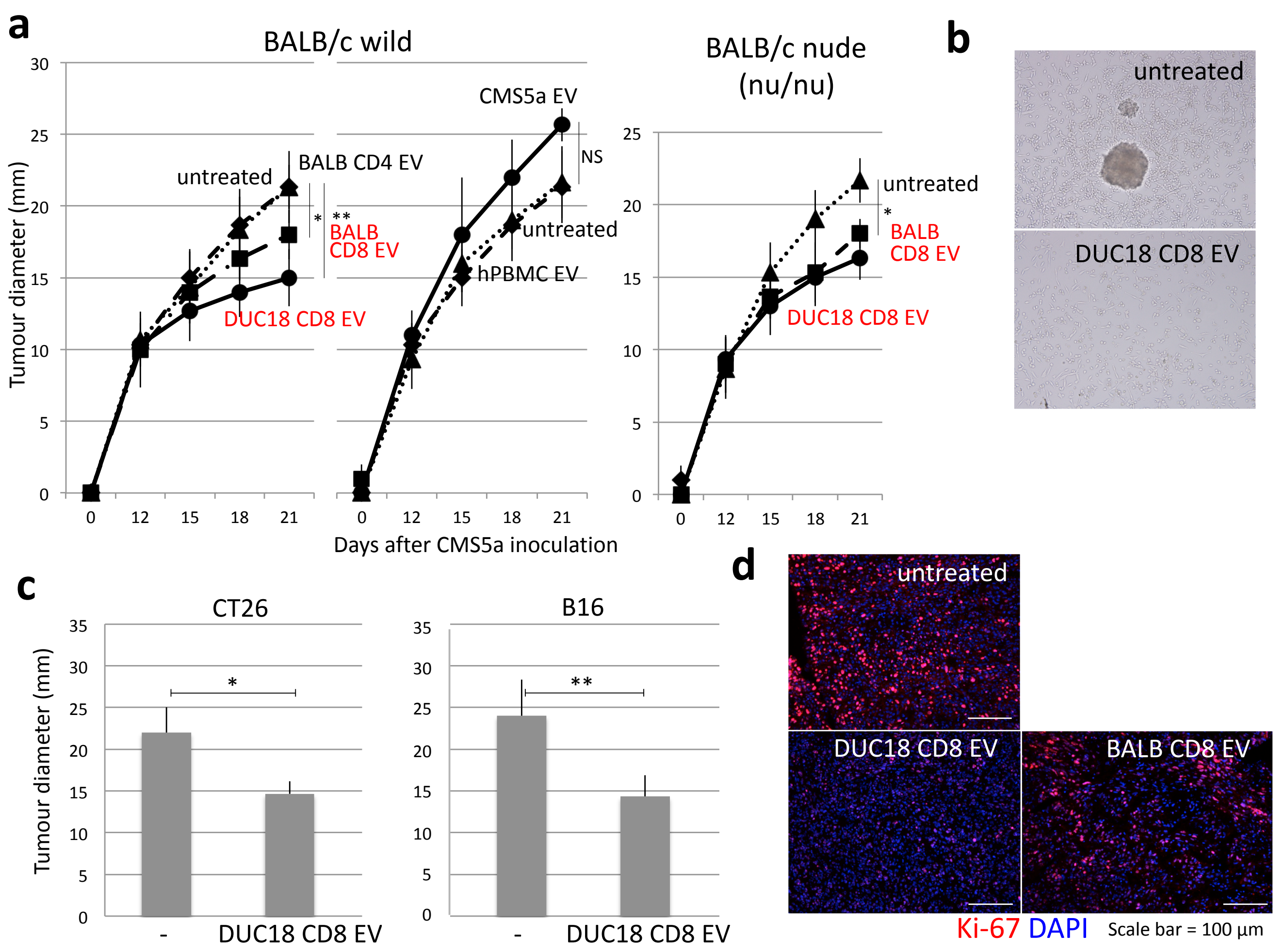

Supplementary Figure 4. Reduction of growth and spheroid-forming capacity of tumours by i.t. treatment of CD8$^+$ T cell EVs. (a) CMS5a-bearing wild-type or nude BALB/c mice were given intratumoural administration (10 μg [5-7 x 10^8 vesicles]/tumour) with DUC18 CD8 EVs, BALB CD8 EVs, BALB CD4 EVs, CMS5a EVs, or hPBMC EVs on day 12, and subcutaneous CMS5a growth was monitored thereafter (*, $p < 0.05$ and **, $p < 0.01$; NS: not significant; error bars indicate SEM). (b) CMS5a tumours at 3 days after treatment with/without DUC18 CD8 EVs were suspended and then cultured for 24 h. Spheroid formation was observed by microscopy. (c) DUC18 CD8 EVs (10 μg [5-7 x 10^8 vesicles]/tumour) were administered to CT26 (BALB/c) or B16 (B6) tumours on day 10 after tumour inoculation, and tumour diameters on day 21 were measured (*, $p <$ 0.05 and **, $p < 0.01$; error bars indicate SEM; n.s.: not significant). The data were analysed by a two-tailed unpaired Student *t*-test. (d) CMS5a tumours at 3 days after the indicated EV treatment were sectioned and stained with Ki-67 mAb (red) and DAPI (blue). Each photo is a representative of 5-6 photos.

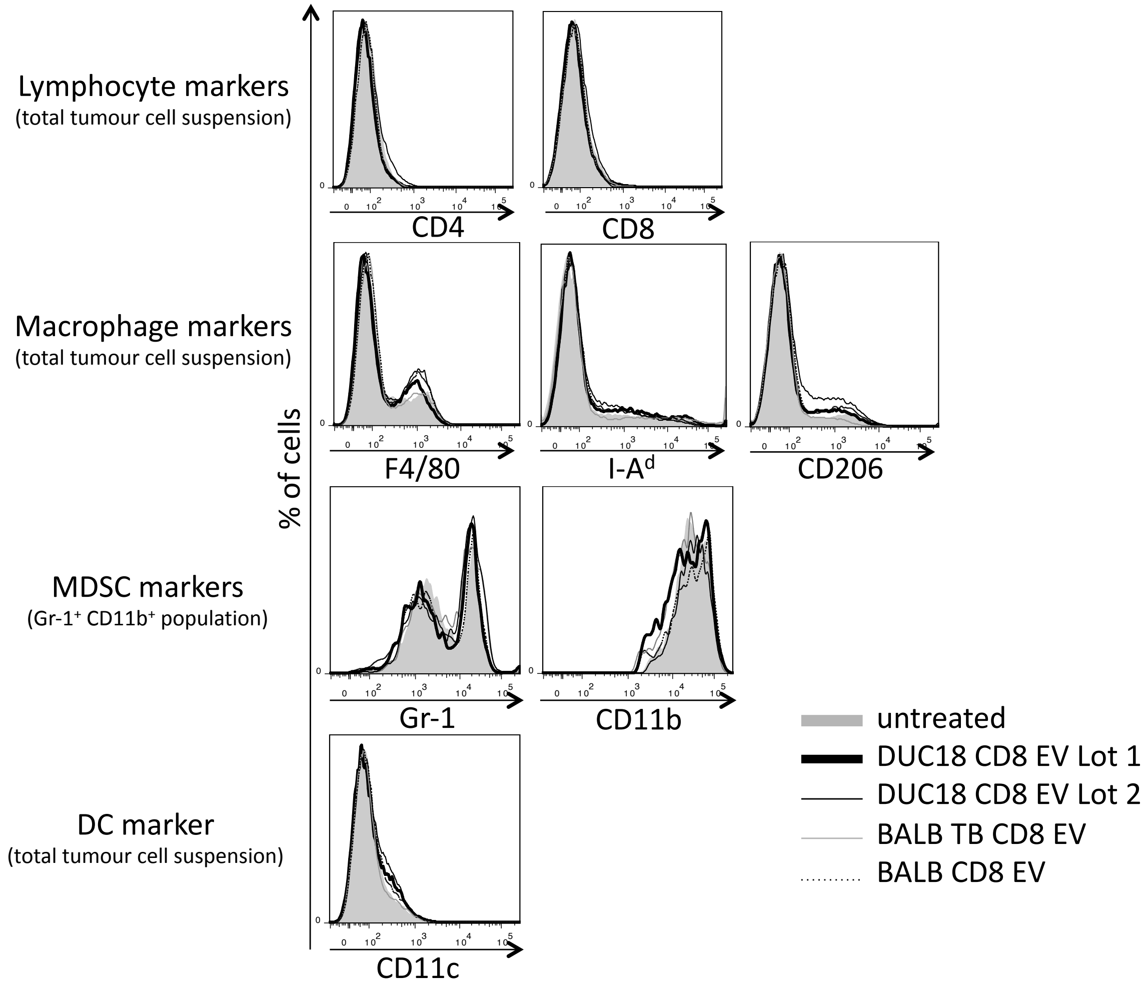

Supplementary Figure 5. Tumour stromal cells other than mesenchymal cells are not affected by CD8$^+$ T cell EV treatment. At 3 days after treatment of subcutaneous CMS5a tumours with/without two lots of DUC18 CD8 EVs, BALB TB CD8 EVs, or BALB CD8 EVs (10 μg [5-7 x 10^8 vesicles]/tumour), obtained tumour cell suspensions were stained with CD4, CD8, F4/80, I-A^d, CD206, Gr-1, CD11b, or CD11c-specific mAb and analysed by flow cytometry.

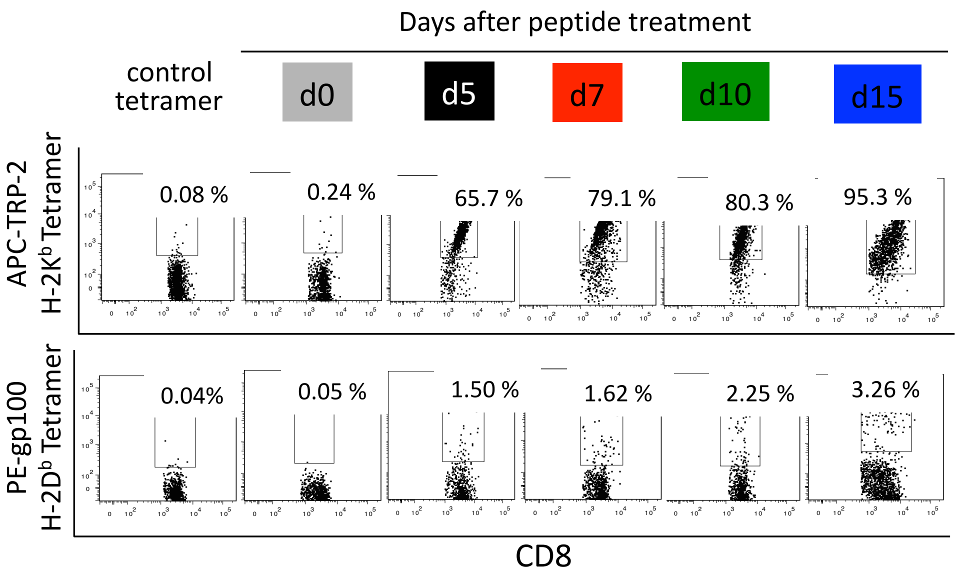

Supplementary Figure 6. B16 melanoma-specific expansion of CD8$^+$ T cells *in vitro*. B6 splenocytes were stimulated with both TRP-2 and gp100 peptides and then monitored for the proportion of TRP-2 or gp100-specific T cells in total CD8$^+$ cells on days 5, 7, 10, and 15 by flow cytometric analysis using the corresponding tetramers (T-select MHC tetramer: MBL Co. Ltd.). An unrelated tetramer was used as a control.

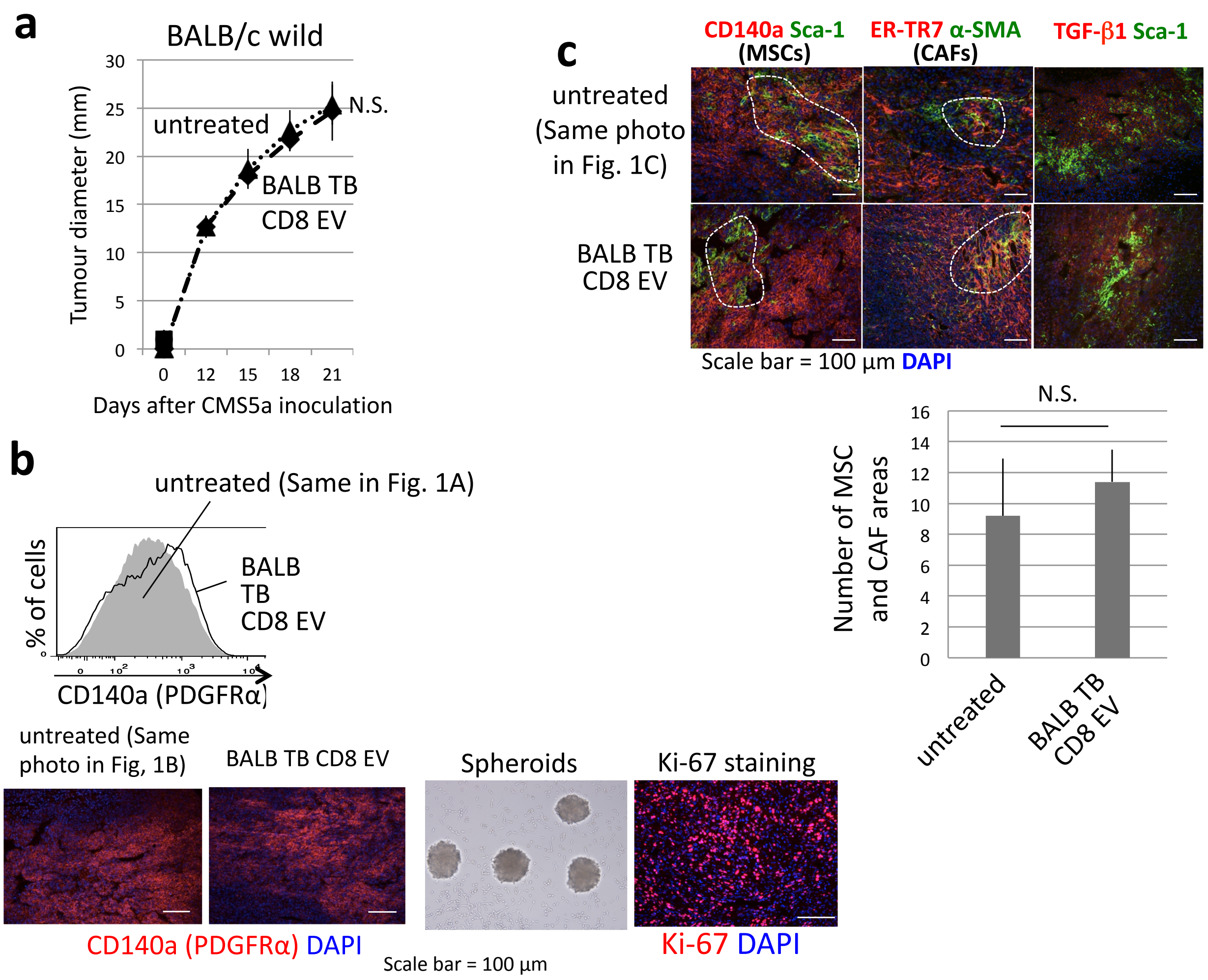

Supplementary Figure 7. No depletion of tumour mesenchymal cells by i.t. treatment with BALB TB CD8 EVs. BALB TB CD8 EVs (10 μg [5-7 x 10^8 vesicles]/tumour) were injected into day 12 CMS5a tumours. Tumour diameters were monitored (n.s., not significant). The data were analysed by a two-tailed unpaired Student *t*-test. (a), and CD140a expression, spheroid formation, Ki-67 expression, MSC area, CAF area, and TGF-β expression were examined by using day 15 tumour sections or suspensions (b, c). CMS5a tumour diameter on day 21 and the number of MSC and CAF areas in the BALB TB CD8 EV-treated group were not significantly different from in the untreated group ($p > 0.01$). The data were analysed by a two-tailed unpaired Student *t*-test.

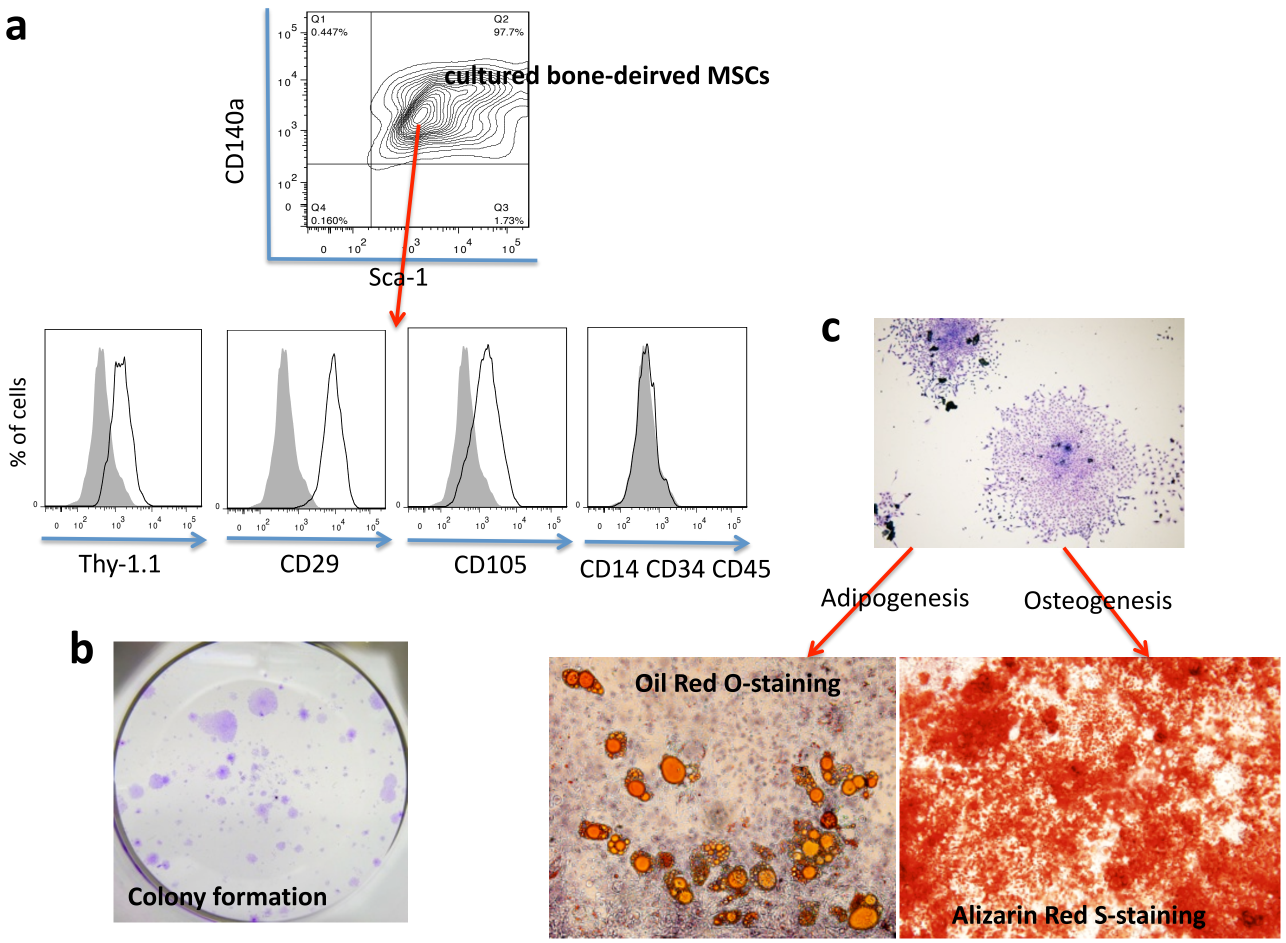

Supplementary Figure 8. Characterization of cultured Thy-1.1^{+} bone-derived MSCs. (a) Thy-1.1^{+} bone-derived MSCs cultured for 1 month were stained with PE-conjugated CD140a and APC-conjugated Sca-1-specific mAbs, and FITC-conjugated Thy-1.1, CD29, or CD105-specific mAb, or a mixture of FITC-conjugated CD14, CD34, and CD45-specific mAbs, and then subjected to flow cytometric analysis. (b) Colony formation of bone-derived MSCs cultured for 2 weeks was visualized by Giemsa staining. (c) Bone-derived MSCs (80% confluent) cultured for 1 month were differentiated with adipogenic or osteogenic medium for 3 weeks. Bone-derived MSCs that differentiated into adipocytes or osteocytes were stained with Oil Red O or Alizarin Red S, respectively.

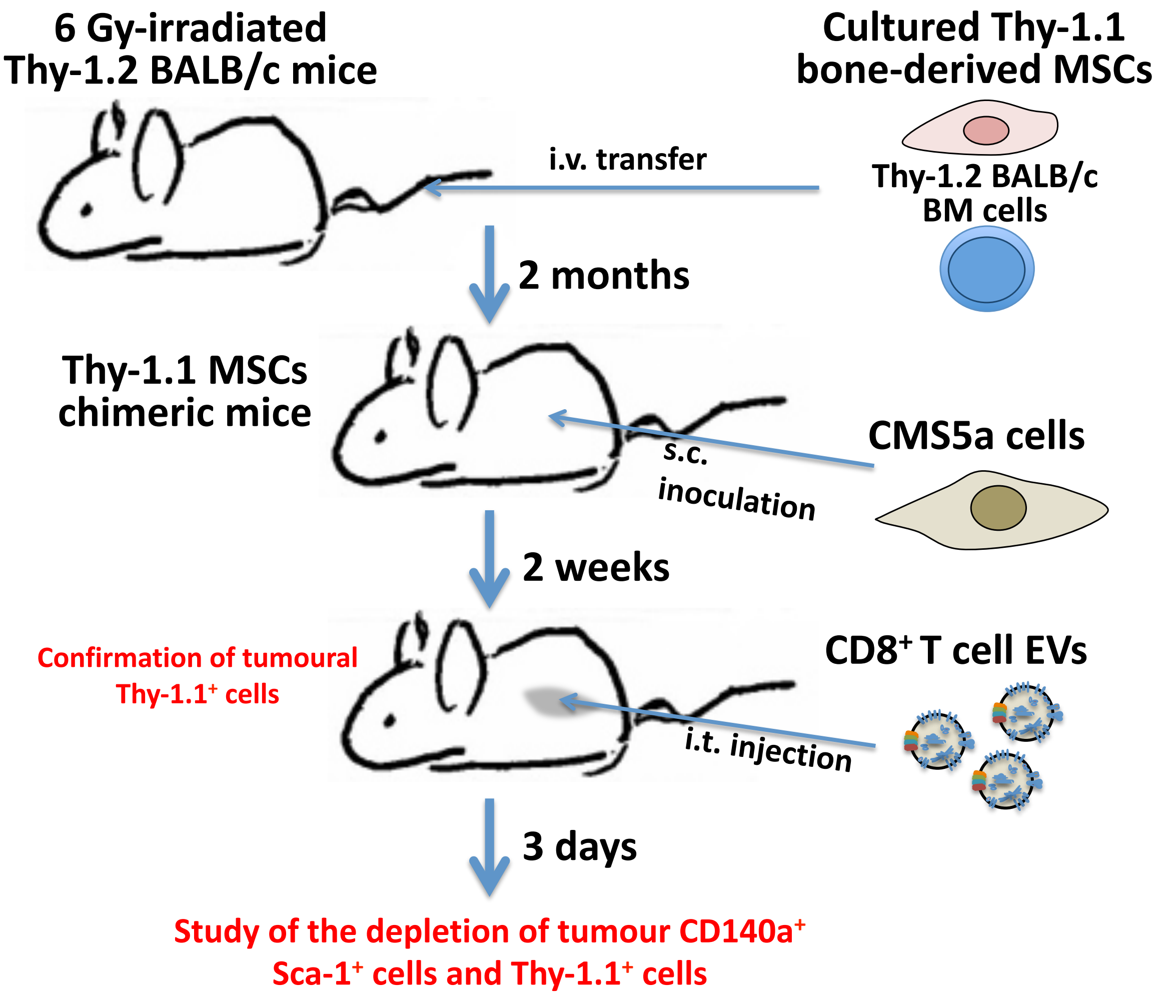

Supplementary Figure 9. Strategy for generation of Thy-1.1^{+} MSC chimeric BALB/c mice. Cultured Thy-1.1^{+} bone-derived MSCs and Thy-1.2^{+} BALB/c bone marrow cells were transferred intravenously into 6 Gy-irradiated BALB/c mice. Two months after transfer, CMS5a cells were inoculated subcutaneously into Thy-1.1^{+} MSC chimeric mice. Two weeks after CMS5a inoculation, DUC18 CD8 EVs or BALB CD8 EVs were injected into CMS5a tumours (approximately 1 cm in diameter).

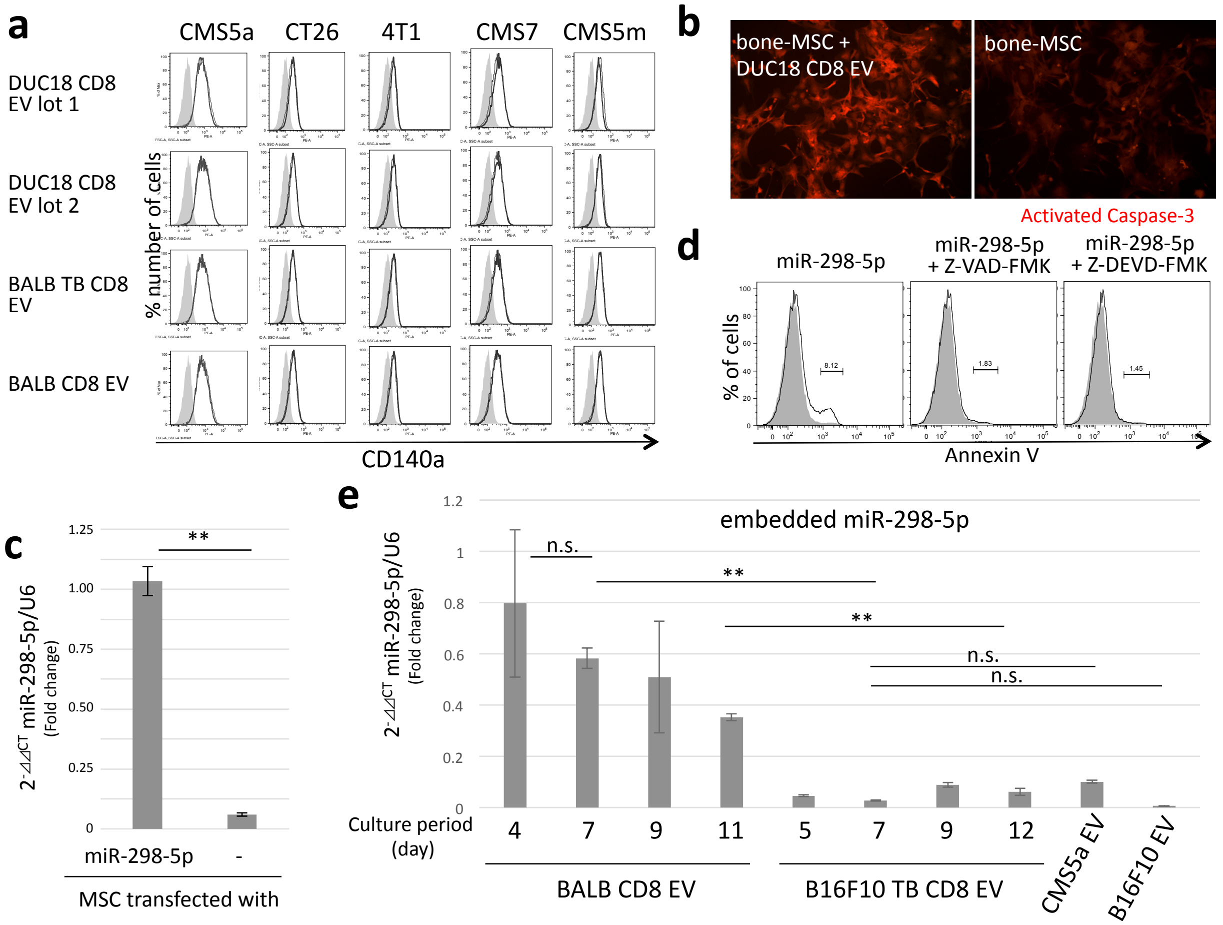

Supplementary Figure 10. Characterization of CD8 EVs and miR-298-5p against tumour cells or MSCs. (a) DUC18 CD8 EVs, BALB CD8 EVs, or BALB TB CD8 EVs were added to the culture of CMS5a, CT26, 4T1, CMS7, or CMS5m (10 μg/mL). Three days after EV treatment, CD140a expression by tumour cells was analysed by flow cytometry. Rat IgG2a mAb was used as a control. (b) Bone-derived MSCs cultured with/without DUC18 CD8 EVs for 2 days were fixed and permeabilized with acetone, and then stained with an activated caspase-3-specific mAb (C92-605: BD Biosciences). (c) Bone-derived MSCs transfected with/without miR-298-5p were subjected to quantitative RT-PCR with miR-298-5p primer at 2 days after transfection (**$p < 0.01$; error bars indicate SEM). The data were analysed by a two-tailed unpaired Student *t*-test. (d) Bone-derived MSCs transfected by miR-298-5p in the presence of pan-caspase inhibitor (Z-VAD-FMK) or caspase-3 inhibitor (Z-DEVD-FMK) were cultured for 3 days, and subjected to flow cytometric analysis after staining with APC-conjugated annexin V. (e) Small RNAs from BALB CD8 EVs or B16F10 TB CD8 EVs obtained from indicated culture periods, CMS5a EVs or B16F10 EVs were subjected to quantitative RT-PCR by using miR-298-5p (**$p < 0.01$; n.s.: not significant; error bars indicate SEM). The data were analysed by a two-tailed unpaired Student *t*-test.

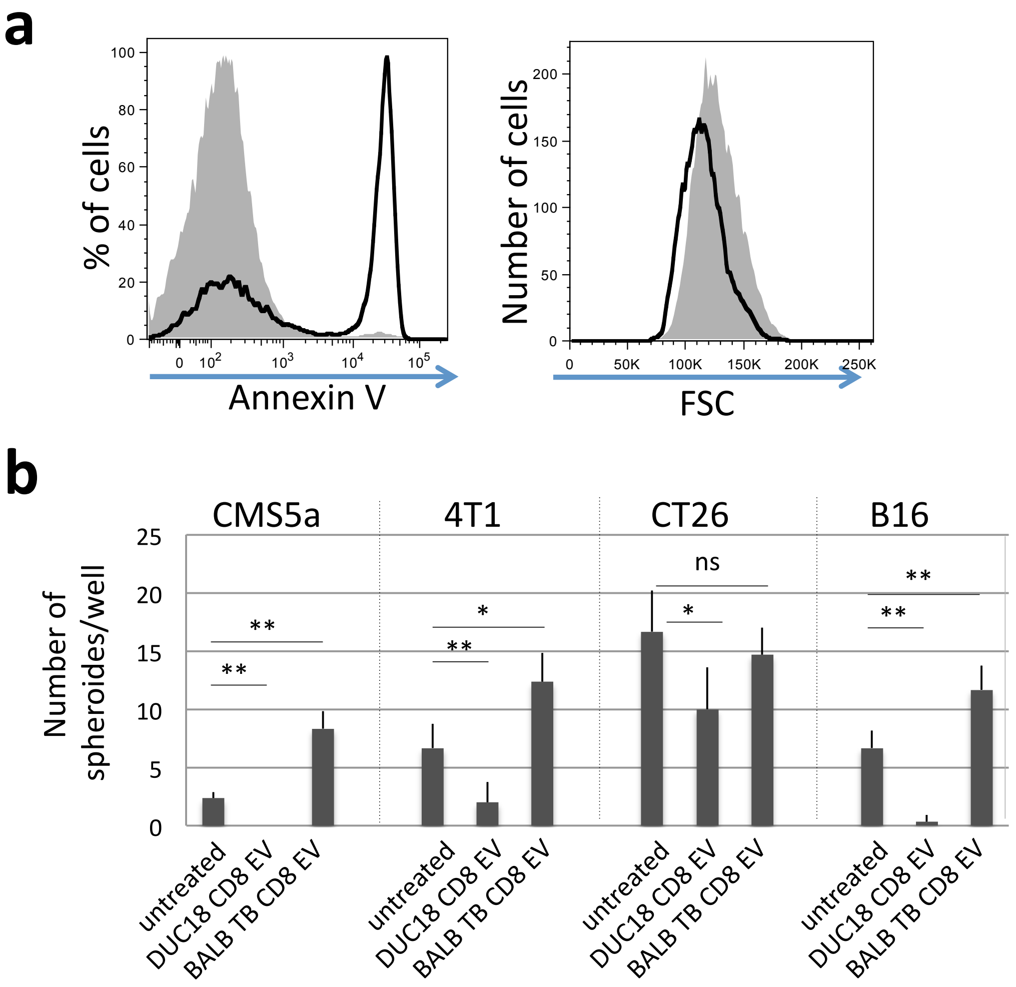

Supplementary Figure 11. Programmed death of Jurkat cells by camptothecin and reduced MSC-mediated spheroid formation of tumour cells by CD8[+] T cell EVs. (a) Jurkat cells treated with 5 μM camptothecin for 5 h were subjected to flow cytometric analysis after staining with annexin V (black line: camptothecin-treated; filled gray: untreated control). (b) The number of spheroids of CMS5a, 4T1, CT26, and B16 cultured with bone-derived MSCs for 4 days in the presence of DUC18 CD8 EVs or BALB TB CD8 EVs were observed microscopically (*, $p < 0.05$ and **, $p < 0.01$; error bars indicate SEM; n.s.: not significant). The data were analysed by a two-tailed unpaired Student t-test.

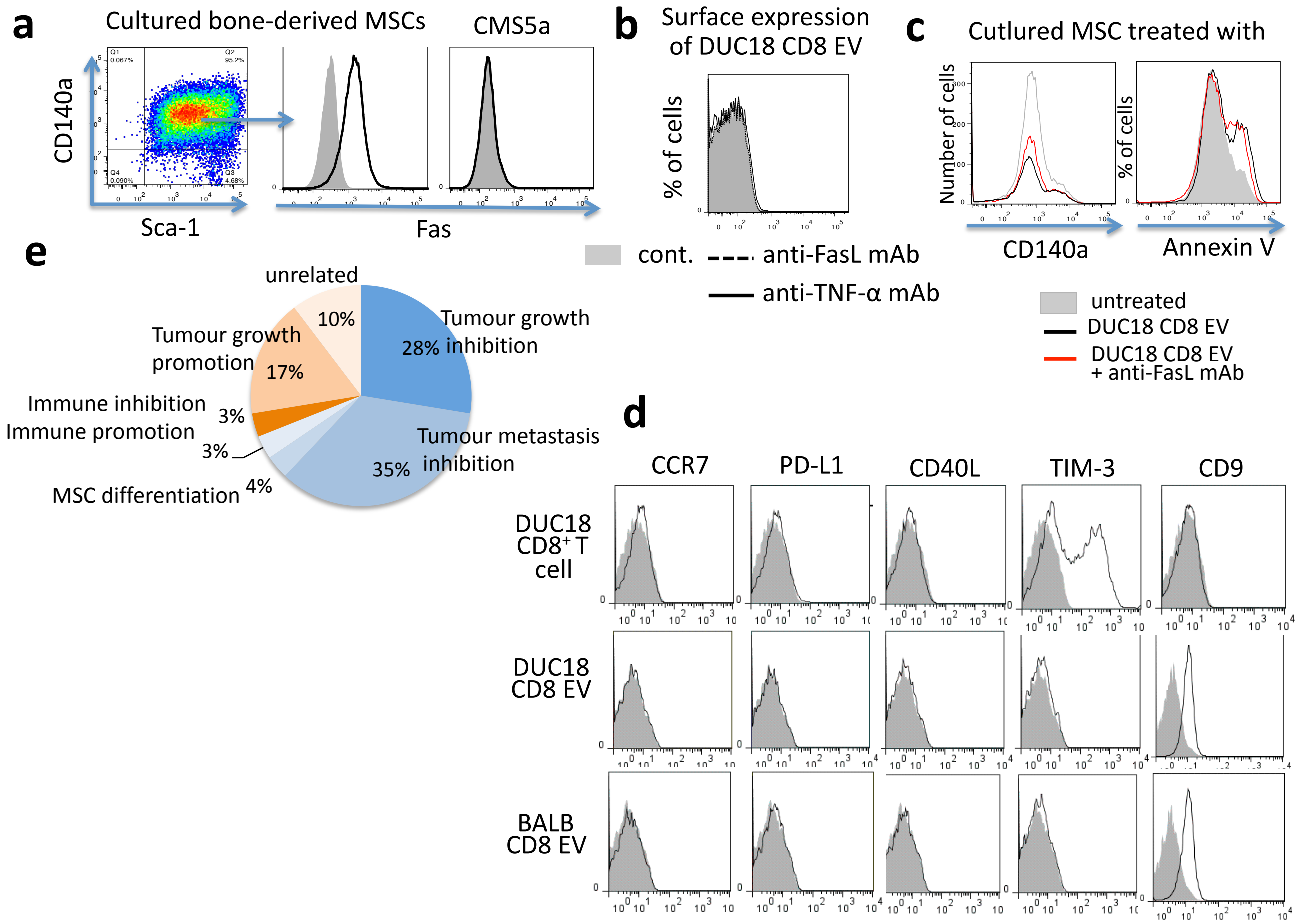

Supplementary Figure 12. No expression of death molecule ligands and T cell markers on CD8^{+} T cell EVs. (a) Bone-derived MSCs or CMS5a cells were stained with Fas-specific mAb (SA367H8: Biolegend) and then subjected to flow cytometric analysis. (b) DUC18 CD8 EVs bound to latex beads were stained with TNF-α (MP6-XT22: Biolegend)- or a FasL (MFL3: Biolegend)-specific mAb. The stained cells were subjected to flow cytometric analysis. (c) Bone-derived MSCs were cultured with DUC18 CD8 EVs with/without FasL-specific mAb (MFL3). After 3 days, the obtained cells were stained with CD140-specific mAb and annexin V, and subjected to flow cytometric analysis. (d) Expression of CCR7, PD-1, CD40L, TIM-3, or CD9 on DUC18 CD8^{+} T cells, DUC18 CD8 EVs, or BALB CD8 EVs were examined by flow cytometry. (e) After a comparison of miRNAs values among DUC18 CD8 EVs, BALB CD8 EVs, and BALB TB CD8 EVs, we selected both DUC18 CD8 EV- and BALB CD8 EV-dominant miRs. The selected miRNAs have been reported to participate in tumour modulation at a high frequency (70–80%).

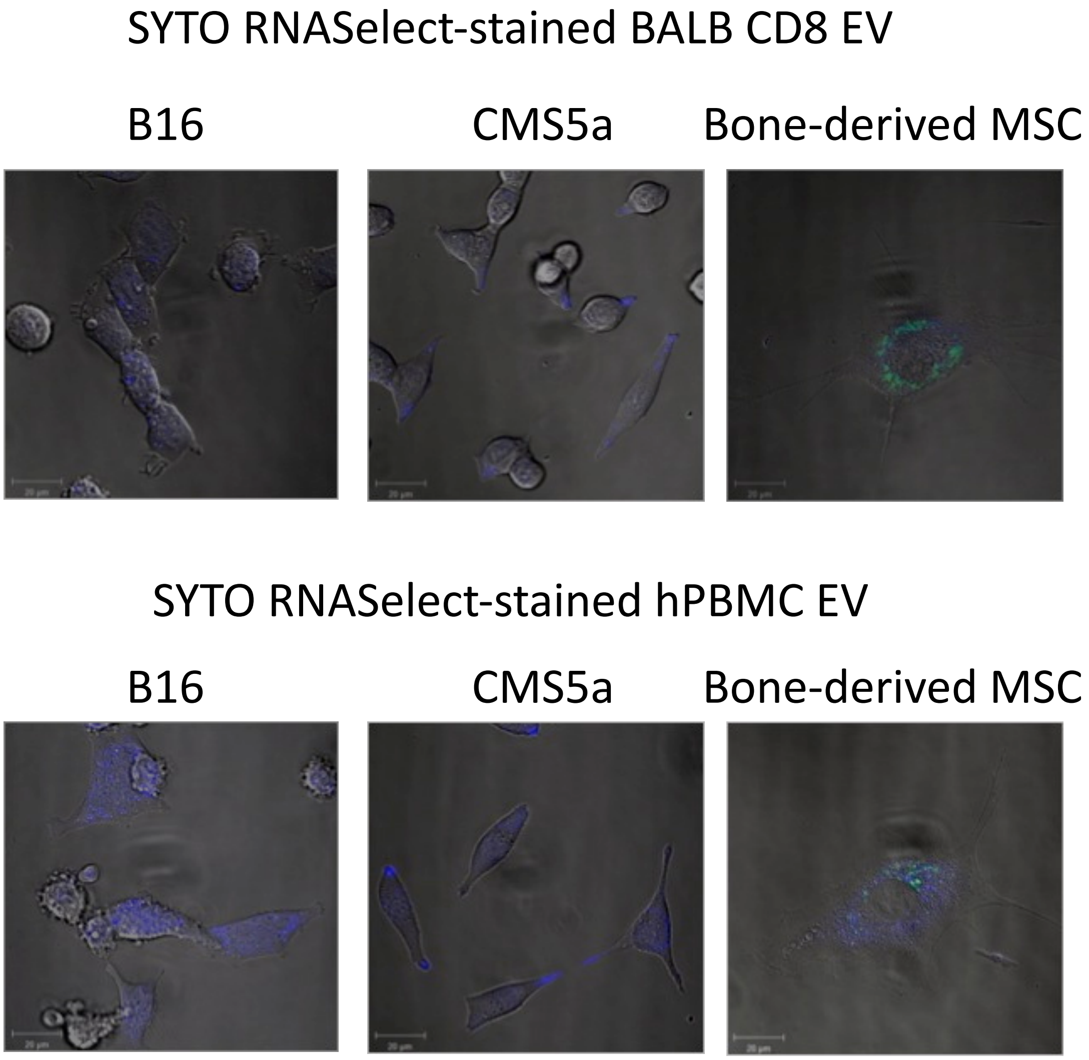

Supplementary Figure 13. Preferential engulfment of mammalian EVs by cultured MSCs. SYTO RNASelect-stained BALB CD8 EV or hPBMC EV was added to B16, CMS5a, or bone-derived MSC cultures. Two hours later, the cells were treated with lysotracker (blue), and then observed by confocal laser scanning microscopy. Each photo is a representative of image of 2-3 photos.

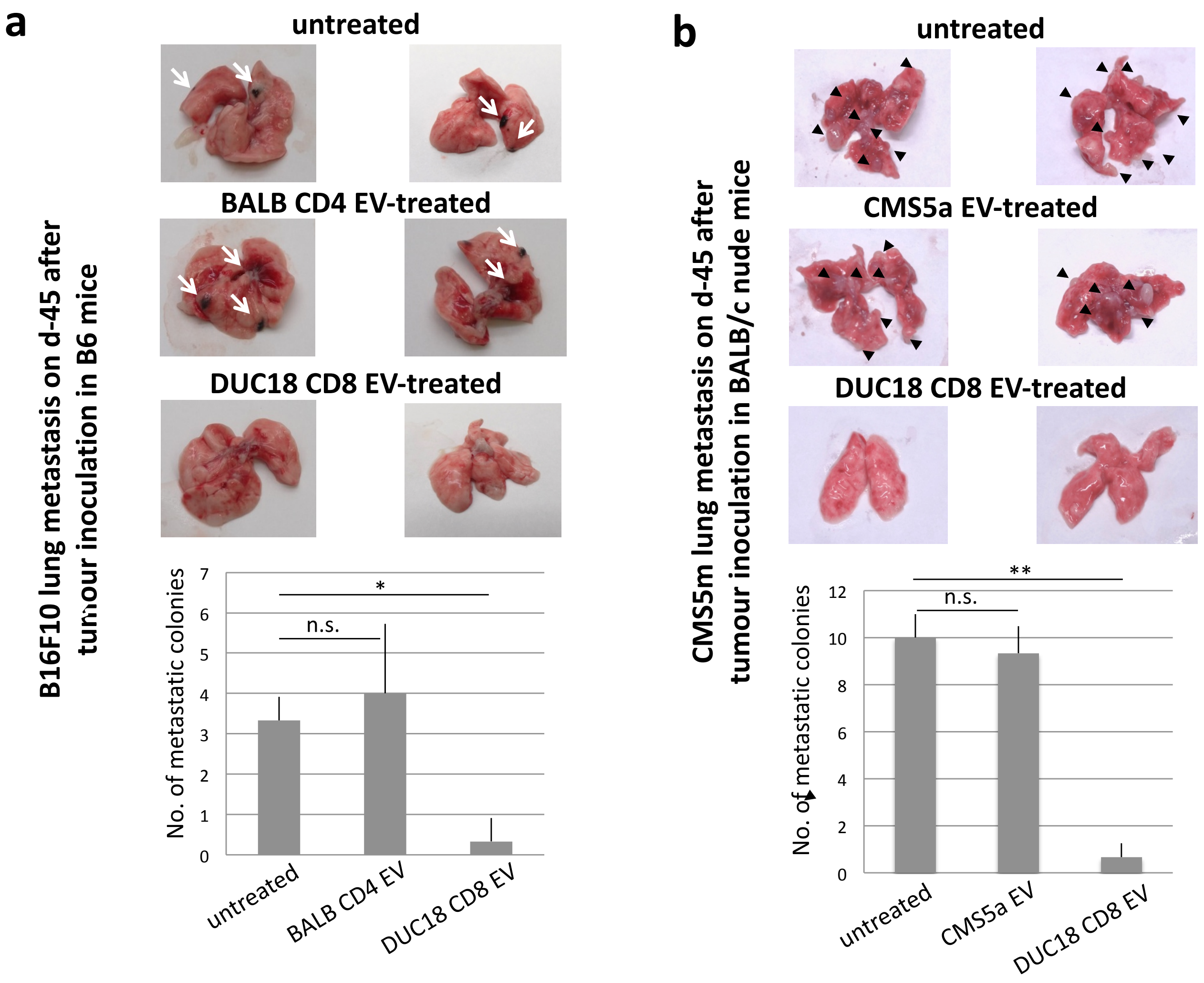

Supplementary Figure 14. No inhibition of tumour lung metastasis by CD4$^+$ T cell EV or endogenous T cells. The protocol for the observation of CMS5m lung metastasis in Fig. 5c was used. Lung metastasis of subcutaneous B16F10 tumours in B6 wild-type mice (a) or subcutaneous CMS5m tumours in BALB/c nude mice (b) on day 45 from the indicated groups was observed. Arrows indicate metastatic colony location of tumours. The numbers of metastatic tumour colonies were counted (*, $p < 0.05$, **, $p < 0.01$, n.s., not significant; error bars indicate SEM). The data were analysed by a two-tailed unpaired Student *t*-test.

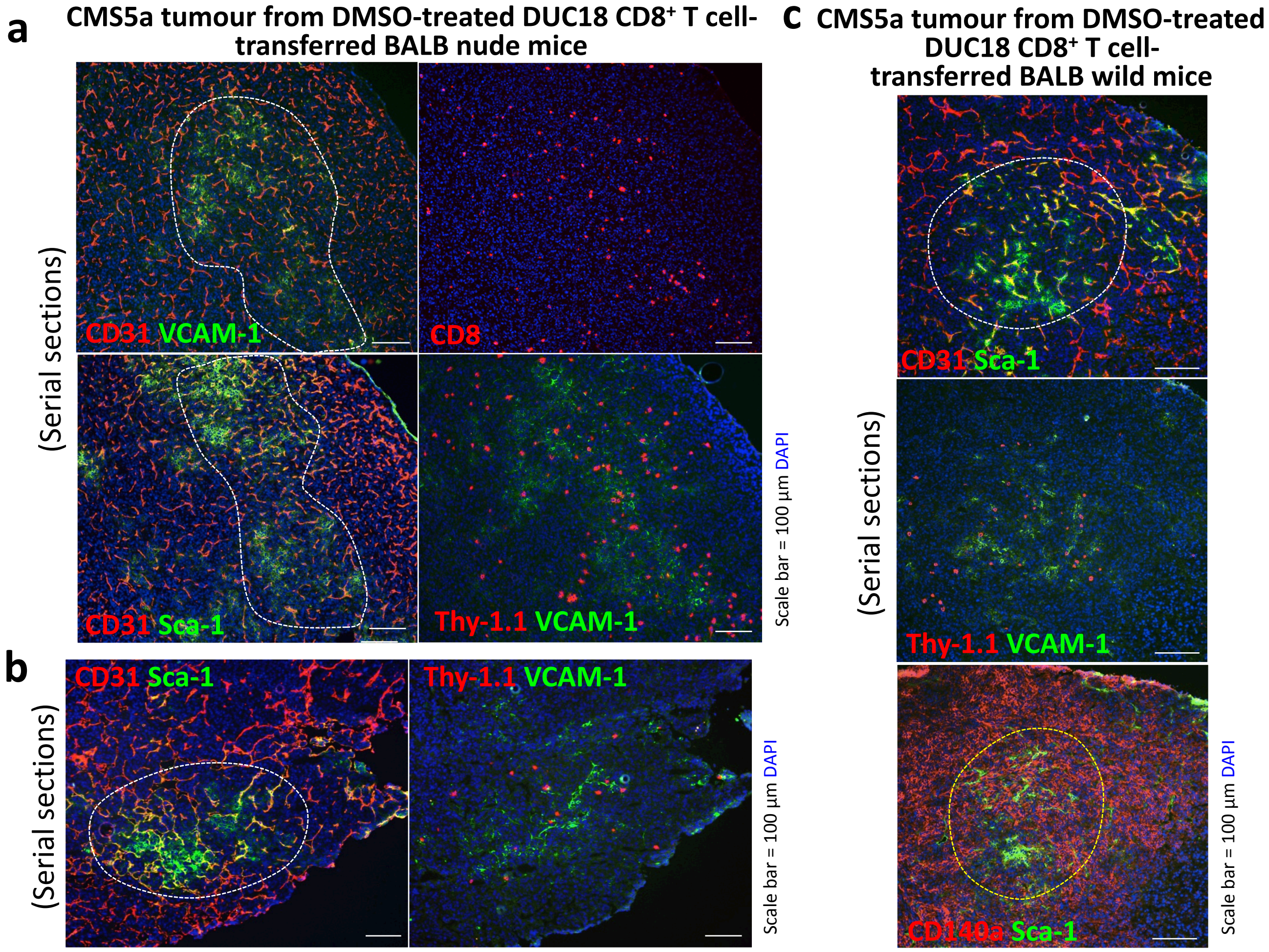

Supplementary Figure 15. Extravasation of adoptive transferred CD8$^+$ T cells at tumour neovascular site. Thy1.1$^+$ DUC18 CD8$^+$ T cells were transferred intravenously into day 12 CMS5a tumour-bearing BALB/c nude (a, b) or wild-type mice (c). The next day, the resected tumours were sectioned and stained with the indicated mAbs and DAPI. White or yellow dotted circles indicate CD31$^+$ VCMA-1$^+$ and CD31$^+$ Sca-1$^+$ neovascularization areas, or CD140a$^+$ Sca-1$^+$ mesenchymal tumour stromal areas, respectively.

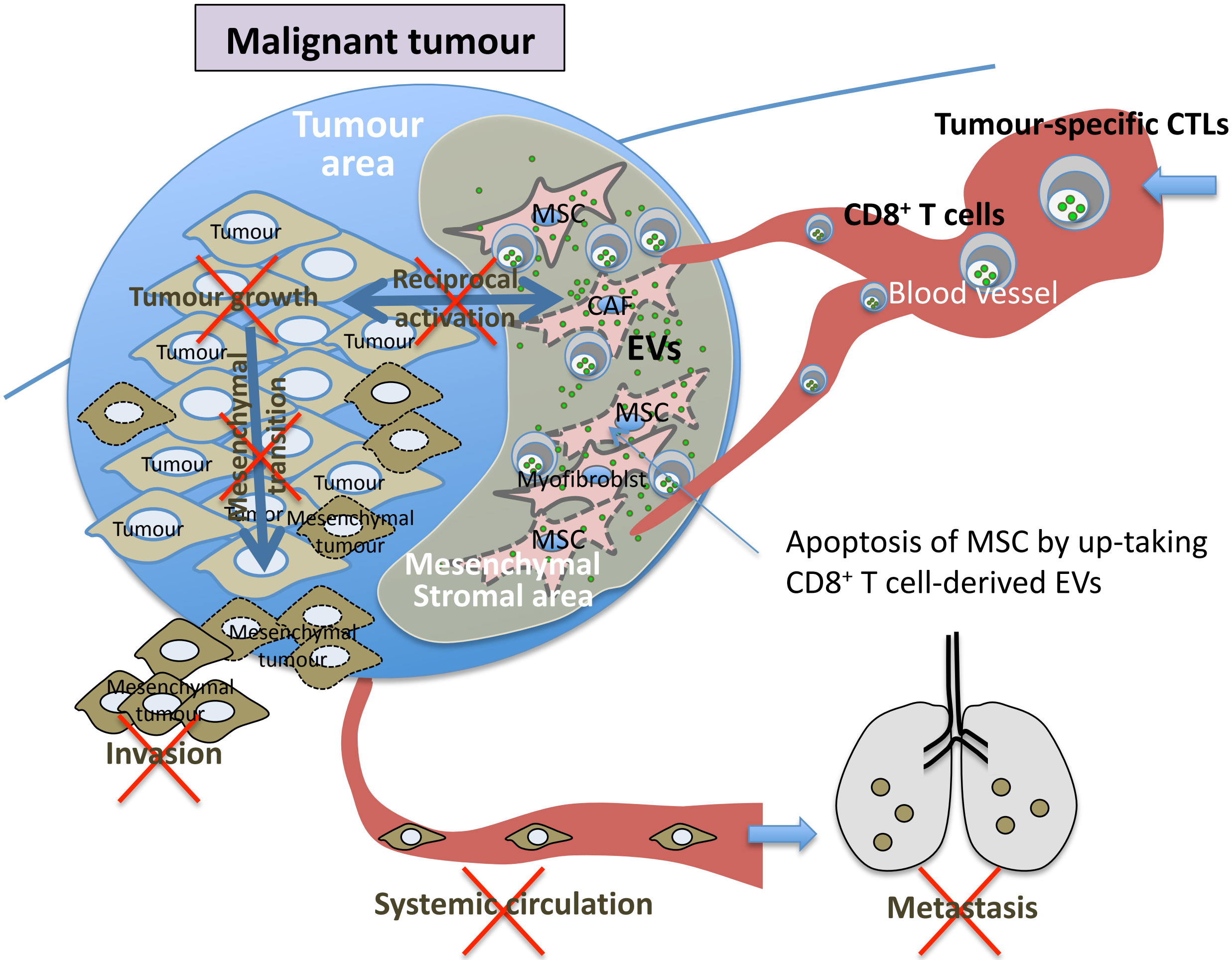

Supplementary Figure 16. Summarized figure regarding function of CD8$^+$ T cell EVs against tumours. CD8$^+$ T cells enter tumour lesions at neovascularization sites, release EVs, and deplete mesenchymal tumour stromal cells by EV-mediated apoptotic death. Depletion of tumour stromal cells including MSCs and CAFs by CD8$^+$ T cell-derived EVs results in reduced tumour growth, and prevention of tumour invasion and metastasis.

miRNA name	DUC18 CD8 EV	BALB CD8 EV	BALB TB CD8 EV	BALB CD4 EV	Bone-derived MSC	CMS5a	B16
miR-298-5p	254.3	147	79.3	94.5	15.9	45	9
miR-351-5p	192.7	86.2	87	85.4	30.1	59.1	36.6
miR-700-3p	169.4	58.5	69.7	48.2	107.4	105.5	49.1
miR-1943-5p	295	107.5	77.3	112.5	34	45.2	38.3
miR-1249-5p	1076.1	879.2	571.4	717.3	70.3	117.2	61.4
miR-370-5p	118.3	32.2	39.4	49.8	n.d.	n.d.	n.d.
miR-6392-5p	114.4	68	55.5	71.0	n.d.	12.9	n.d.
miR-5099	1476.8	475.4	163.7	1198.3	117.8	303.7	221.7
miR-150-5p	58.3	28.1	205.2	172.5	n.d.	n.d.	n.d.
miR-223	43.2	51.4	193.8	70.8	n.d.	7.9	n.d.
miR-491-5p	337.4	537.3	827.9	230.2	19.3	29.3	10.4
miR-1231-5p	65.5	76.4	125.9	72.9	84.3	127.6	123.3
miR-3103-5p	57.8	99.5	166,7	59.1	14.2	26	14.5
miR-3470a	34.3	24.4	79.5	31.3	n.d.	15.8	12.8
miR-3470b	43.2	41.1	101.6	36.6	14.6	26	14.5
miR-6419	28.8	50.2	63.2	48.2	n.d.	n.d.	n.d.

Supplementary Table 1. Normalized raw data of indicated miRNAs in $CD8^+$ T cell EVs and $CD4^+$ T cell EVs, bone-derived MSCs, and tumour cells. Both DUC18 CD8 EV- and BALB CD8 EV-dominant miRNAs over 1.5-fold in the ratio of the global normalized data of corresponding miRNAs from BALB TB CD8 EVs, bone-derived MSCs, or tumour cells are indicated as the filled pale orange. BALB TB CD8 EV-dominant miRNAs over 2.5-fold in the ratio of the global normalized data of corresponding miRNAs from DUC18 CD8 EVs and BALB CD8 EVs are indicated as the filled pale Blue. n.d.: not detected.
